# Global, regional, and national burden of aortic aneurysm disease and its attributable risk factor, 1990–2021: a systematic analysis for the global burden of disease study 2021

**DOI:** 10.1007/s11739-025-04061-8

**Published:** 2025-08-11

**Authors:** Yue Zhuo, Danni Zhao, Mingyao Luo, Zhou Zhou, Chang Shu

**Affiliations:** 1 Center of Vascular Surgery, Fuwai Hospital, Chinese Academy of Medical Sciences & Peking Union Medical College/National Center for Cardiovascular Diseases, State Key Laboratory of Cardiovascular Disease, No. 167, Beilishi Road, Xicheng District, Beijing, 100037 China; 2 Beijing Key Laboratory for Molecular Diagnostics of Cardiovascular Diseases, Center of Laboratory Medicine, Fuwai Hospital, Chinese Academy of Medical Sciences & Peking Union Medical College/National Center for Cardiovascular Diseases, State Key Laboratory of Cardiovascular Disease, Beijing, 100037 China; 3 Department of Vascular Surgery, Fuwai Yunnan Cardiovascular Hospital, Affiliated Cardiovascular Hospital of Kunming Medical University, Kunming, 650102 China; 4 Department of Vascular Surgery, Central China Subcenter of National Center for Cardiovascular Diseases, Henan Cardiovascular Disease Center, Fuwai Central-China Cardiovascular Hospital, Central China Fuwai Hospital of Zhengzhou University, Zhengzhou, 450046 China

**Keywords:** Global burden of disease, Aortic aneurysm, Mortality, Epidemiology

## Abstract

This study aimed to evaluate the global, regional, and national burden of aortic aneurysms and their attributable risk factors from 1990 to 2021. Data from the global burden of disease 2021 study were used to analyze aortic aneurysm death numbers, age-standardized death rates (ASDR), and attributable risk factors across age, sex, region, and the Socio-demographic Index (SDI). Temporal trends were assessed by estimated annual percentage change (EAPC). Pearson correlation analysis evaluated the relationship between disease burden and covariates. In 2021, aortic aneurysm caused 153,927 deaths [95% uncertainty intervals (UI), 138,413–165,739], with an ASDR of 1.86 per 100,000 (95% UI 1.67–2.00). From 1990 to 2021, deaths increased by 74.2% (95% UI 56.4–94.0), while ASDR decreased by 26.8% (95% UI 18.0–34.6) with an EAPC of − 1.28% [95% confidence interval (CI), − 1.38 to − 1.18%]. Male ASDR was 2.25 times higher than females (95% UI 1.90–2.77) and correlated with SDI (*R* = 0.469, *p* < 0.001). The highest ASDR was in the High-income Asia Pacific (4.38 per 100,000; 95% UI 3.72–4.75). Smoking, the leading risk factor, accounted for 30.9% of ASDR (95% UI 26.3–35.8%). Projections estimate that deaths will rise to 174,611 (95% UI 163,289–185,933), with ASDR declining to 1.70 per 100,000 (95% UI 1.45–1.96) in 2030. Aortic aneurysm burden varies by age, sex, and region. Despite declining ASDR, it remains an emerging global public health issue driven by aging populations and shifting risk factors, requiring innovative prevention and healthcare strategies.

**Supplementary Information:**

The online version contains supplementary material available at 10.1007/s11739-025-04061-8.

## Introduction

Aortic aneurysm is defined as a permanent local dilation of the aorta exceeding 50% of the diameter of the adjacent healthy aorta [1]. While most aortic aneurysms are asymptomatic, rupture often leads to fatal outcomes [2]. Early screening and surgical intervention are the best approaches to prevent and manage aortic aneurysms [1, 3, 4]. Therefore, assessing the latest burden of aortic aneurysm and applying effective interventions is crucial.

Most studies on the burden of aortic aneurysms come from epidemiological surveys in developed countries and previous global burden of disease (GBD) studies. Despite advances in prevention, diagnosis, and treatment, the burden remains substantial [5, 6]. Research has linked the burden to the Healthcare Access Quality Index and forecasted future mortality trends [7]. Some well-established cardiovascular risk factors are hypothesized to influence the mortality risk of aortic aneurysms [8, 9]. However, the driving factors behind changes in the burden of aortic aneurysms remain unclear. The role of demographic characteristics, medical advancements, and basic research progress has not been systematically assessed, and regional disparities across geographic and socioeconomic contexts remain insufficiently explained. Additionally, the population-level distribution of risk factors is still unclear, with many related risk factors yet to be thoroughly investigated.

This study utilizes the latest GBD 2021 data to investigate the global mortality burden of aortic aneurysms and examine its trends from 1990 to 2021. The findings are intended to inform clinicians in refining prevention strategies and assist policymakers in making data-driven decisions regarding healthcare resources and public health policies.

## Methods

### Data collection and description

The GBD study, led by the Institute for Health Metrics and Evaluation, estimates global population health trends over time. We used data from the latest GBD 2021 study, accessed via the GBD result tool (https://vizhub.healthdata.org/gbd-results) [10–13].

Aortic aneurysm data included deaths, death rate, age-standardized death rates (ASDR), and attributable risk factors across global, regional, and national levels from 1990 to 2021. The death rate (per 100,000 population) was annual deaths divided by the population. The ASDR (per 100,000 population) was derived by weighting age-specific death rates to a standard population, enabling comparisons across populations with different age structures and over time. Data were stratified by age, sex, year, and geographical hierarchy (seven super-regions, 21 GBD sub-regions, and 204 countries and territories).

### Socio-demographic Index

Data were stratified by the Socio-demographic Index (SDI), a composite indicator from 0 to 1, calculated based on the fertility rate for females under 25, mean education for individuals over 15, and lag-distributed income per capita [14]. Based on annual SDI values, countries and territories were classified into five SDI quintiles (low, low-middle, middle, high-middle, and high) [14]. Higher SDI reflects more remarkable socioeconomic development.

### Temporal trends

Temporal trends in ASDR were evaluated using the estimated annual percentage change (EAPC) from log-linear regression (*y* = *α* + *βx*), with 95% confidence intervals (CI) [15]. A positive EAPC indicated an increase, while a negative EAPC reflected a decline in the ASDR. Joinpoint regression (V.5.2.0) identified trend inflection points using log-scale line fitting and permutation tests, yielding the annual percentage change (APC) and the average APC (AAPC) estimates with 95% CIs [16]. Besides, the auto-regressive integrated moving average (ARIMA) model was applied to forecast the global trend of disease burden through 2030 [17].

### Attributable risk factors

The attributable risk can quantify the burden across risk factors [11]. The GBD 2021 study identified seven attributable risk factors for aortic aneurysm, including smoking, high systolic blood pressure, high body mass index, a diet low in fruits, low in vegetables, high in sodium, and lead exposure. EAPCs for these risks were calculated. Plus, the population attributable fraction (PAF) was calculated and defined as the fraction of all cases that would not have occurred without exposure. The formula for PAF is PAF = (*A*/*O*) × 100%, where *A* is the attributable number of cases, and *O* is the observed number [10–12].

### Statistical analysis

The GBD 2021 study data provided 95% uncertainty intervals (UIs), calculated using 25th- and 975th-ordered draws from 1000 samples from the posterior distribution of each quantity [10, 12]. Pearson’s correlation coefficient was used to examine the relationship between disease burden and covariates. All statistical analyses were conducted using R (V.4.3.3). All *p*-values are based on two-sided tests. A *p*-value less than 0.05 was considered statistically significant.

## Results

### Global level

**Fig. 1:**
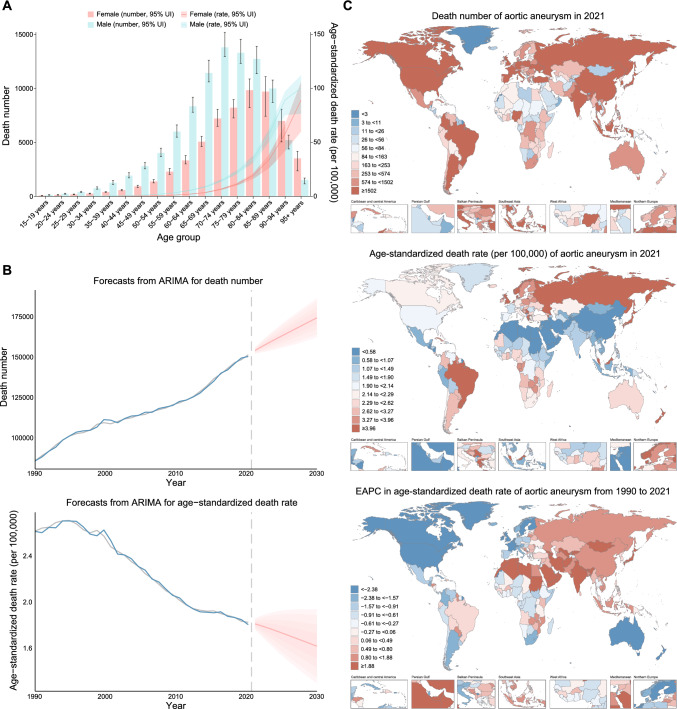
Global burden of aortic aneurysm deaths in 2021 and forecasted trends to 2030. **A** Global age-specific number and age-standardized rate by sex. Error bars indicate the 95% uncertainty interval (UI) for the number. Shading indicates the 95% UI for the rate. **B** Forecasts of number and age-standardized rate to 2030. The gray lines represent estimates for the GBD 2021 study; the blue lines indicate fitted curves by the ARIMA model; the red lines indicate forecasts to 2030 by the ARIMA model, with shaded areas from inside to outside indicating confidence levels of 50, 75, 90, and 95%, respectively. **C** Global burden in 204 countries and territories. *EAPC* estimated annual percentage change, *GBD* global burden of disease study, *ARIMA* auto-regressive integrated moving average

In 2021, an estimated 153,927 deaths (95% UI 138,413–165,739) were attributable to aortic aneurysm, representing a 74.2% increase (95% UI 56.4–94.0) from 88,353 deaths (95% UI 83,090–93,492) in 1990. Despite this, the global ASDR was 1.86 per 100,000 (95% UI 1.67–2.00) in 2021, a 26.8% decrease from 2.54 per 100,000 (95% UI 2.35–2.69) in 1990 (Table 1). Furthermore, in 2021, the number of deaths increased with age, peaking in the 80 to 84 age group. The ASDR for males was about twice that for females (2.01, 95% UI 1.73–2.34), with the rate of increase slowing for males over 95 (Table S1, Fig. 1A).

**Table 1:** Global and regional death number, age-standardized death rate, and EAPC in age-standardized death rate for aortic aneurysm from 1990 to 2021

Location	1990		2021		1990–2021
	Number (95% UI)	Age-standardized rate (per 100,000) (95% UI)	Number (95% UI)	Age-standardized rate (per 100,000) (95% UI)	EAPC (95% CI)
Global	88,353 (83,090 to 93,492)	2.54 (2.35 to 2.69)	153,927 (138,413 to 165,739)	1.86 (1.67 to 2.00)	− 1.28 (− 1.38 to − 1.18)
Female	30,795 (27,622 to 34,388)	1.58 (1.41 to 1.76)	60,063 (51,303 to 66,298)	1.28 (1.10 to 1.42)	− 0.91 (− 1.01 to − 0.81)
Male	57,557 (53,979 to 62,641)	3.87 (3.61 to 4.18)	93,864 (86,610 to 102,153)	2.57 (2.36 o 2.79)	− 1.63 (− 1.74 to − 1.52)
High SDI	53,929 (50,582 to 55,553)	4.76 (4.46 to 4.91)	67,202 (57,735 to 72,287)	2.87 (2.51 to 3.06)	− 1.98 (− 2.11 to − 1.85)
High-middle SDI	18,321 (17,508 to 19,197)	1.99 (1.88 to 2.08)	34,827 (32,309 to 37,274)	1.79 (1.66 to 1.92)	− 0.66 (− 0.82 to − 0.51)
Middle SDI	8804 (8110 to 9844)	1.03 (0.94 to 1.14)	28,528 (25,797 to 30,959)	1.15 (1.04 to 1.25)	0.16 (0.04 to 0.28)
Low-middle SDI	4608 (3664 to 6272)	0.89 (0.71 to 1.20)	16,808 (13,956 to 22,468)	1.31 (1.09 to 1.76)	1.27 (1.21 to 1.33)
Low SDI	2557 (1568 to 4437)	1.37 (0.83 to 2.37)	6371 (3932 to 10,434)	1.48 (0.91 to 2.44)	0.19 (− 0.02 to 0.41)
Central Europe, Eastern Europe, and Central Asia	11,620 (11,257 to 11,991)	2.54 (2.44 to 2.62)	21,530 (20,159 to 22,916)	3.29 (3.08 to 3.5)	0.54 (0.35 to 0.73)
Central Asia	430 (374 to 513)	0.94 (0.81 to 1.13)	1443 (1280 to 1615)	1.98 (1.77 to 2.21)	2.50 (2.26 to 2.73)
Central Europe	4379 (4217 to 4522)	3.07 (2.95 to 3.18)	6682 (6141 to 7318)	2.93 (2.69 to 3.21)	− 0.43 (− 0.61 to − 0.26)
Eastern Europe	6812 (6583 to 7085)	2.52 (2.43 to 2.62)	13,406 (12,354 to 14,430)	3.82 (3.52 to 4.12)	1.00 (0.68 to 1.33)
High-income	57,886 (54,358 to 59,646)	4.68 (4.39 to 4.83)	71,155 (60,945 to 76,598)	2.86 (2.51 to 3.04)	− 1.95 (− 2.10 to − 1.81)
Australasia	1902 (1788 to 2012)	8.03 (7.50 to 8.50)	1549 (1354 to 1680)	2.60 (2.29 to 2.81)	− 4.11 (− 4.27 to − 3.96)
High-income Asia Pacific	5277 (4879 to 5532)	2.79 (2.55 to 2.93)	25,773 (20,940 to 28,524)	4.38 (3.72 to 4.75)	1.57 (1.41 to 1.74)
High-income North America	19,569 (18,209 to 20,354)	5.30 (4.94 to 5.50)	13,970 (12,471 to 14,793)	2.09 (1.89 to 2.20)	− 3.64 (− 3.89 to − 3.39)
Southern Latin America	2121 (1978 to 2296)	4.71 (4.40 to 5.09)	2352 (2167 to 2528)	2.64 (2.43 to 2.83)	− 2.02 (− 2.22 to − 1.81)
Western Europe	29,016 (27,281 to 29,890)	4.78 (4.50 to 4.92)	27,511 (24,098 to 29,189)	2.57 (2.30 to 2.71)	− 2.49 (− 2.73 to − 2.25)
Latin America and Caribbean	5212 (5009 to 5358)	2.53 (2.42 to 2.61)	15,409 (14,146 to 16,375)	2.55 (2.33 to 2.71)	− 0.33 (− 0.51 to − 0.15)
Andean Latin America	193 (164 to 228)	1.00 (0.86 to 1.19)	538 (449 to 644)	0.93 (0.78 to 1.12)	− 0.09 (− 0.22 to 0.05)
Caribbean	921 (851 to 981)	3.79 (3.51 to 4.04)	1405 (1240 to 1583)	2.59 (2.29 to 2.92)	− 1.58 (− 1.73 to − 1.42)
Central Latin America	1198 (1150 to 1240)	1.56 (1.49 to 1.62)	3293 (2865 to 3768)	1.37 (1.19 to 1.56)	− 1.16 (− 1.43 to − 0.89)
Tropical Latin America	2901 (2780 to 2994)	3.36 (3.19 to 3.48)	10,173 (9352 to 10,729)	4.04 (3.70 to 4.26)	0.36 (0.11 to 0.60)
North Africa and Middle East	1059 (776 to 1456)	0.66 (0.49 to 0.89)	3694 (3203 to 4257)	0.89 (0.78 to 1.03)	1.23 (1.07 to 1.38)
South Asia	3457 (2168 to 5573)	0.71 (0.45 to 1.13)	15,979 (11,379 to 23,410)	1.22 (0.88 to 1.78)	1.87 (1.74 to 2.01)
Southeast Asia, East Asia, and Oceania	5055 (4164 to 6171)	0.54 (0.45 to 0.65)	17,707 (15,342 to 20,433)	0.68 (0.59 to 0.79)	0.71 (0.63 to 0.79)
East Asia	2936 (2374 to 3719)	0.36 (0.29 to 0.44)	10,199 (8229 to 12,817)	0.50 (0.41 to 0.63)	1.15 (1.04 to 1.26)
Oceania	50 (38 to 68)	2.29 (1.79 to 2.98)	117 (91 to 151)	1.94 (1.54 to 2.45)	− 0.71 (− 0.80 to − 0.62)
Southeast Asia	2069 (1670 to 2586)	1.03 (0.83 to 1.29)	7391 (6476 to 8513)	1.39 (1.21 to 1.60)	0.79 (0.72 to 0.87)
Sub-Saharan Africa	4063 (2508 to 6457)	2.35 (1.44 to 3.68)	8453 (5029 to 13,139)	2.12 (1.28 to 3.24)	− 0.70 (− 0.85 to − 0.54)
Central Sub-Saharan Africa	476 (263 to 788)	2.69 (1.48 to 4.44)	1057 (591 to 1706)	2.41 (1.35 to 3.88)	− 0.53 (− 0.79 to − 0.27)
Eastern Sub-Saharan Africa	1135 (677 to 1948)	1.82 (1.07 to 3.05)	2636 (1427 to 4293)	1.80 (0.99 to 2.96)	− 0.25 (− 0.43 to − 0.08)
Southern Sub-Saharan Africa	737 (604 to 862)	3.09 (2.49 to 3.66)	1237 (1123 to 1349)	2.46 (2.23 to 2.68)	− 1.36 (− 1.68 to − 1.03)
Western Sub-Saharan Africa	1715 (905 to 3053)	2.36 (1.25 to 4.18)	3523 (1745 to 5980)	2.19 (1.10 to 3.68)	− 0.56 (− 0.71 to − 0.41)

*UI* uncertainty intervals, *EAPC* estimated annual percentage change, *CI* confidence interval

From 1990 to 2021, the global ASDR decreased at an EAPC of − 1.28% (95% CI − 1.38 to − 1.18) (Table 1). Joinpoint analysis identified three inflection points in 1994, 1999, and 2012 (four phases). ASDR showed an upward trend until 1994, after which the trend reversed and began to decline. The most rapid decrease occurred between 1999 and 2012 (Table 2, Table S2, Fig. S1A). To be more specific, ASDR declined in all age groups except those over 95, with the most significant reductions in the 70–79 age groups. In contrast, the ASDR for over 95 increased by 7.5%, with an EAPC of 0.22% (95% CI 0.15–0.29).

**Table 2:** Temporal trend of age-standardized death rate for aortic aneurysm by sex from 1990 to 2021

	Segment 1		Segment 2		Segment 3		Segment 4		AAPC (95% CI)
	Years	APC (95% CI)	Years	APC (95% CI)	Years	APC (95% CI)	Years	APC (95% CI)	
Both	1990–1994	0.803** (0.471 to 1.137)	1994–1999	− 0.674** (− 1.003 to − 0.344)	1999–2012	− 1.842** (− 1.910 to − 1.774)	2012–2021	− 0.708** (− 0.831 to − 0.585)	− 0.987** (− 1.064 to − 0.910)
Male	1990–1995	0.414* (0.138 to 0.690)	1995–2003	− 1.647** (− 1.807 to − 1.487)	2003–2013	− 2.288** (− 2.403 to − 2.173)	2013–2021	− 0.824** (− 0.976 to − 0.672)	− 1.314** (− 1.390 to − 1.237)
Female	1990–1994	0.883* (0.331 to 1.439)	1994–1999	− 0.112 (− 0.648 to 0.428)	1999–2011	− 1.523** (− 1.643 to − 1.403)	2011–2021	− 0.459** (− 0.609 to − 0.309)	− 0.645** (− 0.768 to − 0.522)

*APC* annual percent change, *AAPC* average annual percent change, *CI* confidence interval

*Significantly different from 0 (*p* < 0.01)

**Significantly different from 0 (*p* < 0.001)

By 2030, the global deaths from aortic aneurysms are projected to reach 174,611 (95% UI 163,289–185,933). However, despite this increase, the global ASDR for aortic aneurysms is expected to decline to 1.70 per 100,000 (95% UI 1.45–1.96) (Fig. 1B).

### Regional and national level

In 2021, significant regional differences were observed in the global ASDR for aortic aneurysms. High ASDR was seen in Central Europe, Eastern Europe, Northern Asia, and Eastern South America (primarily Brazil and surrounding areas). At the same time, lower ASDR was observed in Eastern Asia and Northern Africa, predominantly in lower-latitude regions (Table 1; Fig. 1C). At the super-region level, Central Europe, Eastern Europe, and Central Asia had the highest ASDR at 3.29 per 100,000 (95% UI 3.08–3.50), while Southeast Asia, East Asia, and Oceania recorded the lowest at 0.68 per 100,000 (95% UI 0.59–0.79). Regionally, the High-income Asia Pacific region had the highest ASDR, reaching 4.38 per 100,000 (95% UI 3.72–4.75). At the national level, Armenia recorded the highest ASDR globally (9.16 per 100,000; 95% UI 7.61–10.81), while Saudi Arabia had the lowest (0.21 per 100,000; 95% UI 0.16–0.28) (Table 1, Table S3).

The ASDR showed age-related increases in all regions, peaking in the 90–94 age group (Fig. S1B). ASDR for males was generally higher than for females across the seven global regions. In Eastern Europe, the male-to-female ASDR ratio was highest, at 3.00 (95% UI 2.56–3.52). At the country level, for instance, San Marino's ratio reached 8.47 (95% UI 4.36–16.46). However, in some countries like the United Arab Emirates in the North Africa and Middle East region, the female ASDR surpassed the male ASDR (ratio 0.32; 95% UI 0.21–0.48). Sex differences were prominent in high-income regions and Eastern Europe, with less disparity in parts of Asia and Africa (Table S4, Fig. S2).

From 1990 to 2021, the ASDR declined in the High-income, Latin America and Caribbean, and Sub-Saharan Africa regions, while increasing in South Asia, North Africa and Middle East, Southeast Asia, East Asia and Oceania, and Central Europe, Eastern Europe, and Central Asia. The greatest decline occurred in the High-income region (EAPC − 1.95; 95% CI − 2.10 to − 1.81), and the greatest increase in South Asia (EAPC 1.87; 95% CI 1.74–2.01) (Table 1).

### SDI level

**Fig. 2:**
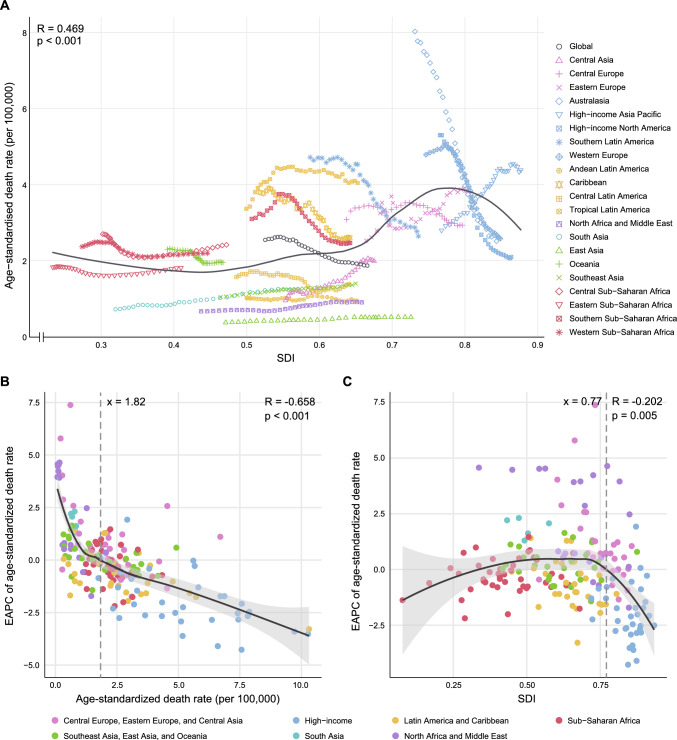
Global trends and correlations of age-standardized death rate of aortic aneurysm from 1990 to 2021. **A** The age-standardized rate for 21 GBD regions by SDI from 1990 to 2021. The expected age-standardized rate in 2021 based solely on SDI was represented by the gray line. The points from left to right depict estimates for each year from 1990 to 2021. **B** The correlation between EAPC in age-standardized rate from 1990 to 2021 and age-standardized rate in 1990. **C** The correlation between EAPC in age-standardized rate from 1990 to 2021 and SDI in 2021. The relationship curve was represented by the black line. Shading indicates the 95% confidence intervals for the EAPC. The R and p values were derived from Pearson correlation analysis. *GBD* global burden of disease study, *SDI* Socio-demographic Index, *EAPC* estimated annual percentage change

The burden of aortic aneurysm was heavier in high-SDI regions, while it is relatively lower in low-SDI regions (Table 1, Table S5). Figure 2A illustrates the relationship between ASDR and SDI across regions, comparing observed ASDR with expected levels based on SDI. Pearson correlation analysis revealed a significant positive correlation between SDI and ASDR (*r* = 0.469, *p* < 0.001). Specifically, the expected ASDR levels varied across SDI categories. In low-SDI regions, the expected ASDR slightly decreased as SDI rose. In middle-SDI regions, it gradually increased, whereas in high-SDI regions, the expected ASDR decreased with rising SDI. Notably, in some regions, ASDR trends were utterly opposite to expectations. For instance, in the High-income Asia Pacific region, ASDR significantly increased with rising SDI. In contrast, in middle-SDI regions like Southern Latin America, ASDR significantly decreased with increasing SDI, similar to high-SDI regions.

### The association between ASDR, SDI, and EAPC

As shown in Fig. 2B, C, the EAPC of ASDR for aortic aneurysm was significantly correlated with the 1990 ASDR and 2021 SDI. The ASDR in 1990 represented the baseline disease burden, while the SDI in 2021 reflected healthcare access and health standards. A significant negative correlation was found between EAPC and ASDR in 1990 (*r* = − 0.58, *p* < 0.001), indicating that a higher baseline burden led to a slower or even declining growth in ASDR, with acceleration in decline. Additionally, when SDI exceeded 0.77, a significant negative correlation between EAPC and SDI (*r* = − 0.33, *p* < 0.001) was observed, suggesting that higher SDI countries experienced faster reductions in aortic aneurysm ASDR from 1990 to 2021. This correlation was absent below an SDI of 0.77, highlighting the role of advanced healthcare systems in high-SDI countries.

### Attributable risk factors

In 2021, approximately 66,078 (95% UI 57,283–74,600) global deaths from aortic aneurysm were attributable to known risk factors, accounting for 42.93% (95% UI 36.13–49.72%) of total deaths. Among the seven risk factors analyzed, smoking was the leading cause, with a PAF of 30.9% (95% UI 26.3–35.8%), followed by high systolic blood pressure (PAF 17.4%; 95% UI 13.1–22.0%) and high body mass index (PAF 7.49%; 95% UI 4.07–12.9%) (Table 3).

**Table 3:** The population attributable fraction of risk factors for aortic aneurysm from 1990 to 2021

Risk factor	Population attributable fraction (PAF) (95% UI)			
	1990	2000	2010	2021
Smoking	41.3 (35.3 to 47.5)	38.1 (32.4 to 44.0)	34.3 (29.1 to 39.8)	30.9 (26.3 to 35.8)
High systolic blood pressure	20.3 (15.5 to 25.3)	19.1 (14.5 to 23.9)	17.2 (13.0 to 21.8)	17.4 (13.1 to 22.0)
High body-mass index	7.27 (3.97 to 12.2)	7.46 (4.02 to 12.6)	7.05 (3.80 to 12.0)	7.49 (4.07 to 12.9)
Diet low in fruits	4.26 (2.94 to 5.63)	3.96 (2.72 to 5.23)	3.68 (2.53 to 4.86)	3.64 (2.49 to 4.81)
Diet low in vegetables	3.53 (2.37 to 4.98)	3.16 (2.12 to 4.48)	2.88 (1.92 to 4.08)	2.87 (1.91 to 4.04)
Diet high in sodium	0.701 (0.0652 to 2.21)	0.764 (0.0701 to 2.41)	0.879 (0.0807 to 2.73)	0.885 (0.0806 to 2.75)
Lead exposure	0.551 (− 0.0736 to 1.45)	0.610 (− 0.0811 to 1.59)	0.679 (− 0.0903 to 1.77)	0.658 (− 0.0895 to 1.72)

*UI* uncertainty intervals

**Fig. 3:**
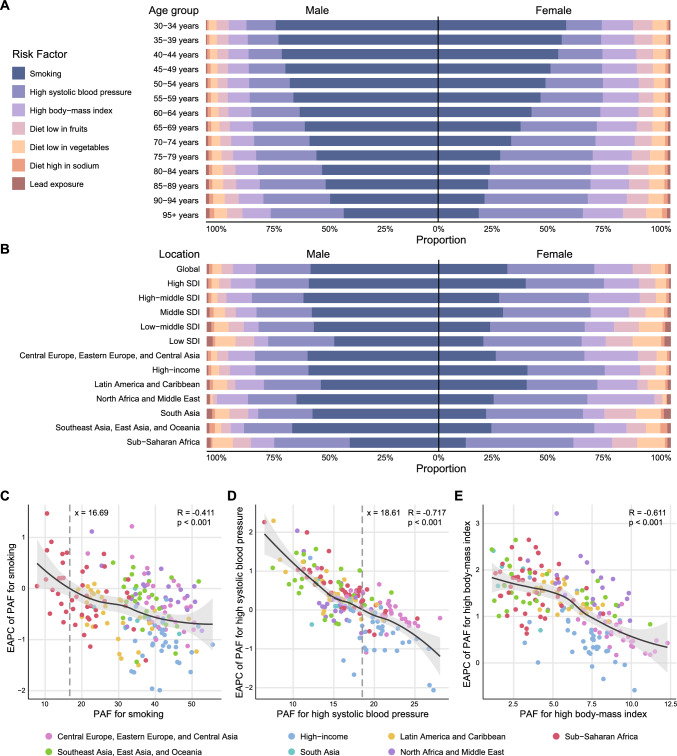
Global patterns of risk factor attribution for aortic aneurysm deaths in 2021. **A** Proportions of age-standardized death rates accounted for risk factors for both sexes by age in 2021. **B** Proportions of age-standardized death rates accounted for risk factors for both sexes by region in 2021. **C** The correlation between EAPC in major PAF from 1990 to 2021 and major PAF in 1990 cross regions. Major PAF of aortic aneurysm includes smoking, high systolic blood pressure, and high body mass index. *SDI* Socio-demographic Index, *EAPC* estimated annual percentage change, *PAF* population attributable fraction

The impact of these risk factors varied across sex, age, and region. Smoking-related ASDR was the highest in males, at 1.01 per 100,000 (95% UI 0.84–1.18), while high systolic blood pressure led female ASDR at 0.23 per 100,000 (95% UI 0.17–0.30). From ages 35–95, the smoking-attributable ASDR increased with age, though its relative proportion decreased. Meanwhile, high systolic blood pressure showed a marked increase in both ASDR and proportion across all age groups as age advances, suggesting that the relative importance of high systolic blood pressure in aortic aneurysm mortality risk became more prominent with age (Fig. 3A). Additionally, there were significant differences in smoking-related mortality across regions with varying SDI regions. Smoking-related ASDR significantly increased with rising SDI levels in both males and females (Fig. 3B).

Figure 3C–E and Fig. S3 showed a significant negative correlation between PAF and EAPC for smoking, high systolic blood pressure, and high body mass index, indicating initial success in the traditional risk factors control. In contrast, EAPC for low fruit intake, low vegetable intake, high sodium intake, and lead exposure showed an initial decrease followed by a slight increase as PAF increases, highlighting the need to refine control strategies for these risk factors.

## Discussion

### Epidemiological trends

Based on GBD 2021 data, this study reports on the latest global burden and temporal trends of aortic aneurysms, revealing a significant rise in deaths but a decline in ASDR. Recent data from the UK National Abdominal Aortic Aneurysm Screening Programme support these findings, demonstrating a sustained reduction in population-level aneurysm mortality over the past decade [18], further corroborating the global trends identified in this analysis.

The burden of aortic aneurysm varies significantly across age groups. In 2021, global deaths due to aortic aneurysms totaled 153,927, with 118,489 (76.98%) occurring in individuals aged over 65. This trend corresponds with a 48% increase in the global population and a 137% rise in those aged over 65 [14], suggesting that the increase in deaths is primarily attributable to demographic shifts (population growth and aging) rather than changes in disease pathophysiology (Table S6). The age group with the highest mortality in 2021 was slightly older than in 1990, further reflecting the impact of population aging. Age-related vascular changes, such as endothelial dysfunction, smooth muscle cell phenotypic switching, extracellular matrix remodeling [19, 20], and cumulative exposure to comorbid cardiovascular conditions (e.g., hypertension and atherosclerosis), may contribute to elevated aneurysm mortality in older populations [21, 22]. Also, poorer surgical tolerance among elderly patients further increases procedural risks [23].

Sex differences in aortic aneurysm burden are also notable. In individuals aged 15–89, male deaths consistently exceed those of females, whereas females surpassed males after age 90, likely due to longer females’ life expectancy [24]. Males exhibit higher ASDR across regions, particularly in Eastern Europe (male-to-female ASDR ratio 3.0; 95% UI 2.56–3.52), with extreme values observed in San Marino (8.47; 95% UI 4.36–16.46). Similarly, recent data from China reported that males accounted for approximately 79.4% of hospitalized aortic aneurysm patients in 2022, further supporting the male predominance observed in our analysis [25]. This pattern aligns with prior evidence identifying the male sex as a key biological risk factor for aortic aneurysm development [26]. Therefore, targeted screening and early intervention strategies remain essential, particularly for aging male populations at the highest risk.

### Regional and socioeconomic disparities

Significant regional disparities were observed in the burden of aortic aneurysms. ASDRs increased progressively with SDI level. This pattern may partly reflect variations in healthcare accessibility, screening implementation, and treatment capacity across socioeconomic levels [27]. In high-income countries, widespread population-based screening, earlier diagnosis, and improved elective surgical management have likely contributed to lower aneurysm mortality. Conversely, broader screening and more sensitive practices may also elevate the reported burden in high-SDI regions by detecting previously unrecognized cases. In contrast, in low-income regions, limited availability of screening programs and vascular surgical services may lead to underdiagnosis, delayed intervention, and worse outcomes, thereby contributing to underestimated disease burden [28]. ASDR deviations from SDI expectations in certain regions suggest additional complex contributors may also exist, warranting further investigation.

Interestingly, a significant negative correlation was observed between the EAPC in ASDR from 1990 to 2021 and baseline ASDR levels in 1990. Regions and countries with lower baseline ASDR tend to experience rising mortality, while those with higher baseline ASDR see slower growth or even a decline. In low-ASDR baseline countries, aortic aneurysms are often deprioritized. These patterns may reflect varying prioritization of non-communicable disease control and disparities in healthcare resource allocation, particularly in low-SDI regions where limited capacity for early detection and intervention remains a major barrier.

In addition to healthcare disparities, environmental factors (e.g., climate, seasonal variation, and atmospheric pressure) [29, 30] and genetic disorders (such as Marfan syndrome, Loeys-Dietz syndrome, and vascular Ehlers-Danlos syndrome) [31] may contribute to regional heterogeneity. However, these influences are likely secondary to systemic differences in healthcare capacity.

### Attributable risk factors and cross-disease evidence

According to GBD 2021 data, smoking holds an absolute dominant position in risk factors for aortic aneurysm mortality, contributing the highest proportion among all assessed risk factors. Epidemiological studies consistently demonstrate that current smokers face markedly elevated risks compared to former or never smokers, with dose–response relationships linked to smoking intensity, duration, and time since cessation [32, 33]. Hypertension is another key factor, with sustained high systolic blood pressure accelerating aortic dilatation and rupture risk [34].

In population subgroups, smoking-related mortality is more prominent in males, whereas high systolic blood pressure contributes more substantially to the mortality burden in females and older adults. With advancing age, the absolute mortality attributable to both smoking and high systolic blood pressure rises; however, the relative contribution of smoking gradually declines while that of high systolic blood pressure increases, particularly in older females.

Socioeconomic disparities further influence risk factor burden. Smoking-attributable ASDR is significantly higher in high-SDI regions compared to low-SDI regions. Although smoking prevalence has declined in high-SDI regions, current ASDR levels remain elevated due to the lagged effects of historical smoking exposure [35].

Notably, aortic aneurysm, lower extremity peripheral arterial disease, ischaemic heart disease, and ischaemic stroke share several common risk factors and have exhibited broadly consistent trajectories in risk factor-attributable burden from 1990 to 2021. PAF for smoking and high systolic blood pressure have shown a declining trend, whereas those for elevated BMI and high sodium intake have gradually increased despite their relatively minor contributions to the overall burden (Table 3, Table S7). These parallel risk factor trajectories, together with the sustained declines in age-standardized mortality reported for multiple cardiovascular and cerebrovascular diseases [36–38], primarily reflect the cumulative impact of global risk factor control strategies. For aortic aneurysms, which lack effective disease-specific preventive interventions, improvements in shared risk factors likely play a predominant role in burden reduction. Additionally, reductions in competing risks from other cardiovascular conditions may have further contributed by extending life expectancy and increasing the time window for aneurysm development.

### Public health and clinical implications

International guidelines for aortic aneurysms emphasize the importance of early identification and management of modifiable risk factors to reduce mortality. Interventions targeting tobacco cessation, blood pressure control, and obesity management have been widely incorporated into both primary and secondary prevention strategies for aortic aneurysms. Declining EAPC trends observed in several regions, along with significant negative correlations between PAFs and corresponding EAPCs, suggest substantial global progress in controlling key risk factors and stabilizing disease burden in high-risk populations [35]. These improvements may partially result from global health initiatives such as the World Health Organization Framework Convention on Tobacco Control and national hypertension management programs [39]. Nevertheless, substantial disparities persist across regions. Low- and middle-SDI regions face ongoing challenges in risk factor control, health education, and access to preventive interventions. Future global health policies should reduce exposure to modifiable risk factors and expand population-level access to prevention and behavioral interventions, potentially generating significant long-term public health benefits.

These findings also offer important implications for clinical practice. Given the progressive and largely asymptomatic nature of aortic aneurysms, systematic screening of high-risk individuals remains critical, especially older adults and those with a history of smoking or hypertension. Clinicians should prioritize smoking cessation and blood pressure management as part of routine cardiovascular care for high-risk patients to reduce aneurysm progression, prevent rupture, and improve long-term outcomes. In resource-limited regions, where surgical interventions are often scarce, improving access to diagnostic imaging, such as abdominal ultrasound and computed tomography angiography, may facilitate earlier identification and better risk stratification. Furthermore, advances in surgical techniques and perioperative management, particularly the adoption of minimally invasive approaches like endovascular aneurysm repair, are essential to improving survival outcomes, especially among elderly patients with higher surgical risks [40, 41].

Additional consideration should be given to the potential impact of the COVID-19 pandemic on aneurysm care. Pandemic-related disruptions in screening, delays in elective procedures, and changes in health-seeking behavior may have contributed to deferred diagnoses and increased rupture events [42–45]. These findings emphasize the importance of ensuring screening and clinical management continuity during public health crises to avoid delayed detection and adverse outcomes.

### Limitation

However, this study has several limitations. First, regional disparities in GBD data about the mortality burden of aortic aneurysm quality merit attention. In low-SDI regions, constrained healthcare infrastructure and inadequate risk factor surveillance may compromise the validity of epidemiological estimates. Sudden fatalities from undiagnosed aneurysm rupture may be misclassified as unspecified cardiovascular events in regions with limited autopsy rates, creating systematic underreporting that skews mortality assessments. Second, data timeliness, like update delays, may introduce temporal biases that distort outcome interpretation. Third, the relatively short projection horizon to 2030 may limit the assessment of long-term trends and policy effects, but extended projections were constrained by data availability and model stability. Moreover, the GBD study relies on assumptions and modeling methods, and all model-based estimates are yet to be determined. Finally, the lack of differentiation between thoracic and abdominal aortic aneurysms may obscure specific epidemiological characteristics, thereby limiting accuracy.

## Conclusion

In conclusion, the age-standardized rates of deaths from aortic aneurysms have declined globally, but the total number of deaths continues to rise with population aging, with a significantly higher burden on males than females. Low- and middle-SDI regions face healthcare resource limitations, contrasting with high-SDI areas benefiting from improved risk control and treatment. In regions with the same SDI level, trends in disease burden correlate with their baseline levels. In addition, effective management of populations at high risk is critical. Smoking remains the leading attributable risk factor, particularly among men, followed by high systolic blood pressure, which is especially prevalent in women and older adults. This study provides the latest valuable insights into the global burden of aortic aneurysms, providing evidence for policymakers to tailor interventions based on regional and demographic disparities (Fig. S4).

## Supplementary Information

Below is the link to the electronic supplementary material.

Supplementary file1 (PDF 17041 KB)

Supplementary file2 (DOCX 120 KB)

## Data Availability

Data resources were available online from the Global Health Data Exchange query tool (https://ghdx.healthdata.org/gbd-results-tool).

## Abbreviations

GBD: Global burden of disease
ASDR: Age-standardized death rates
SDI: Socio-demographic Index
EAPC: Estimated annual percentage change
CI: Confidence interval
APC: Annual percentage change
AAPC: Average APC
ARIMA: Auto-regressive integrated moving average
PAF: Population attributable fraction
UI: Uncertainty intervals
