# Supplementary material for: Global, regional, and national burden of aortic aneurysm disease and its attributable risk factor, 1990–2021: a systematic analysis for the global burden of disease study 2021
Source: Intern Emerg Med. 2025 Aug 11;20(7):2089–101. doi: 10.1007/s11739-025-04061-8 (PMC12534316; doi:10.1007/s11739-025-04061-8)

Internal and Emergency Medicine

Global, Regional, and National Burden of Aortic Aneurysm Disease and Its Attributable Risk Factor, 1990–2021: A Systematic Analysis for the Global Burden of Disease Study 2021

Supplemental Material： Supplemental Figures

Yue Zhuo[1,#], Danni Zhao[2,#], Mingyao Luo[1,3,4,*], Zhou Zhou[2,**], Chang Shu[1,***]

Chang Shu, email address: changshu@vip.126.com; address: No.167, Beilishi Road, Xicheng District, Beijing, China.

1	**Supplemental figure titles and legends**
2	**Fig. S1** Joinpoint regression analysis of the global age-standardized death rate by sex from 1990 to 2021
3	**Fig. S2** Sex ratio (male to female) of age-standardized death rate of aortic aneurysm in 204 countries and territories in 2021
4	**Fig. S3** The correlation between EAPC in minor PAF from 1990 to 2021 and minor PAF in 1990 cross regions
5	**Fig. S4** Graphical summary of the key findings on the latest global burden of aortic aneurysm

Supplemental figure titles and legends

Fig. S1 Joinpoint regression analysis of the global age-standardized death rate by sex from 1990 to 2021

(A) Joinpoint regression analysis of the global age-standardized death rate by sex from 1990 to 2021. (B) The age-standardized death rate by age, GBD super-regions, and 21 GBD regions in 2021. GBD, Global Burden of Disease.

Fig. S2 Sex ratio (male to female) of age-standardized death rate of aortic aneurysm in 204 countries and territories in 2021

Fig. S3 The correlation between EAPC in minor PAF from 1990 to 2021 and minor PAF in 1990 cross regions

Minor PAF includes a diet low in fruits (A), a diet low in vegetables (B), a diet high in sodium (C), and lead exposure (D). EAPC, estimated annual percentage change; PAF, population attributable fraction.

Fig. S4 Graphical summary of the key findings on the latest global burden of aortic aneurysm

ASDR, age-standardized death rate; 65y, 65-year-old; C.Europe, Central Europe; E.Europe, Eastern Europe; C.Asia, Central Asia; SE.Asia, Southeast Asia; E.Asia, East Asia; SDI, Socio-demographic Index; Mid, middle; High-mid, high-middle; Mid-low, middle-low; PAF, population attributable fraction, defined as the fraction of all deaths that would not have occurred without exposure; SBP, systolic blood pressure.

Supplemental Material: Fig. S1

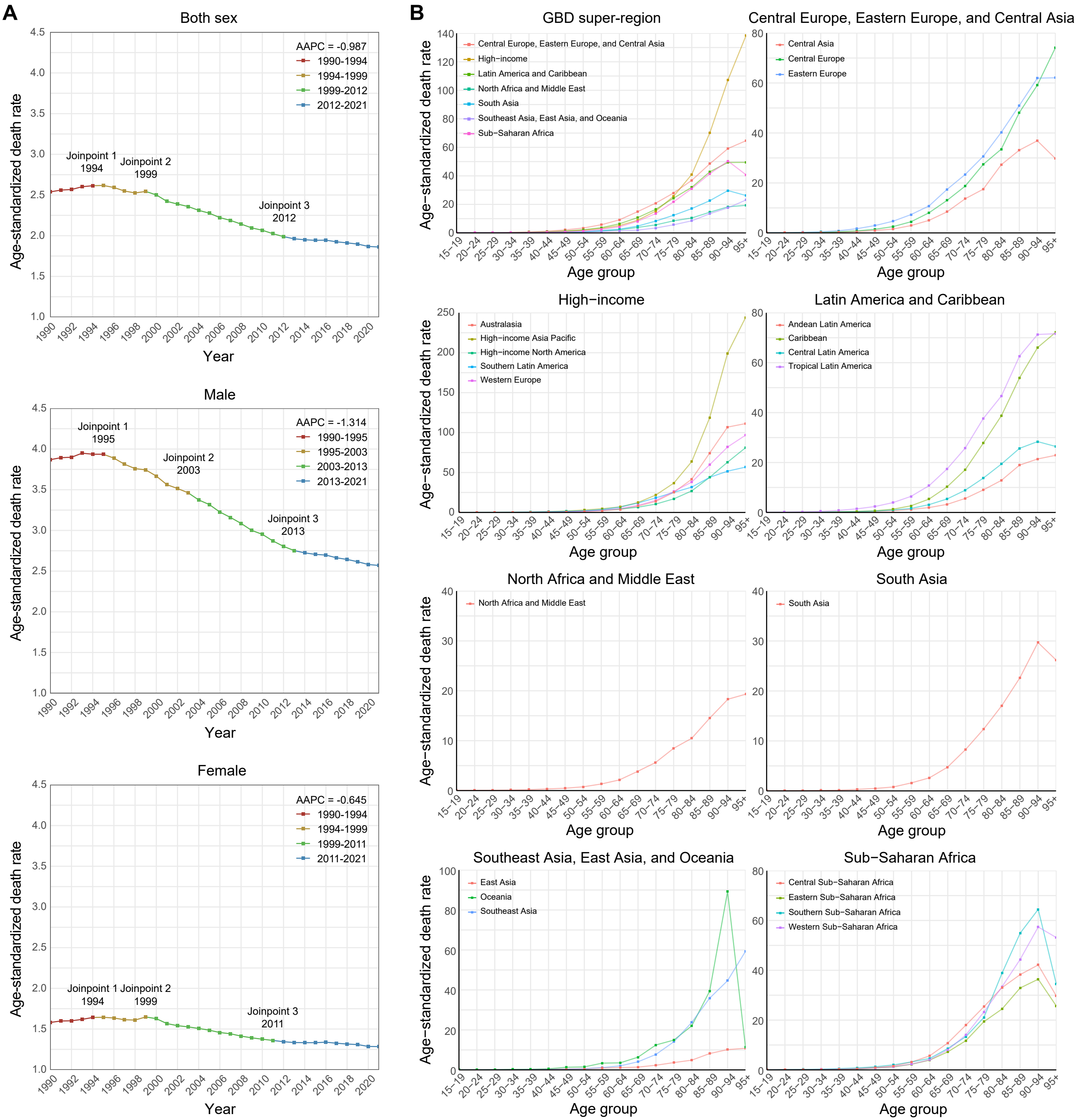

Supplemental Material: Fig. S2

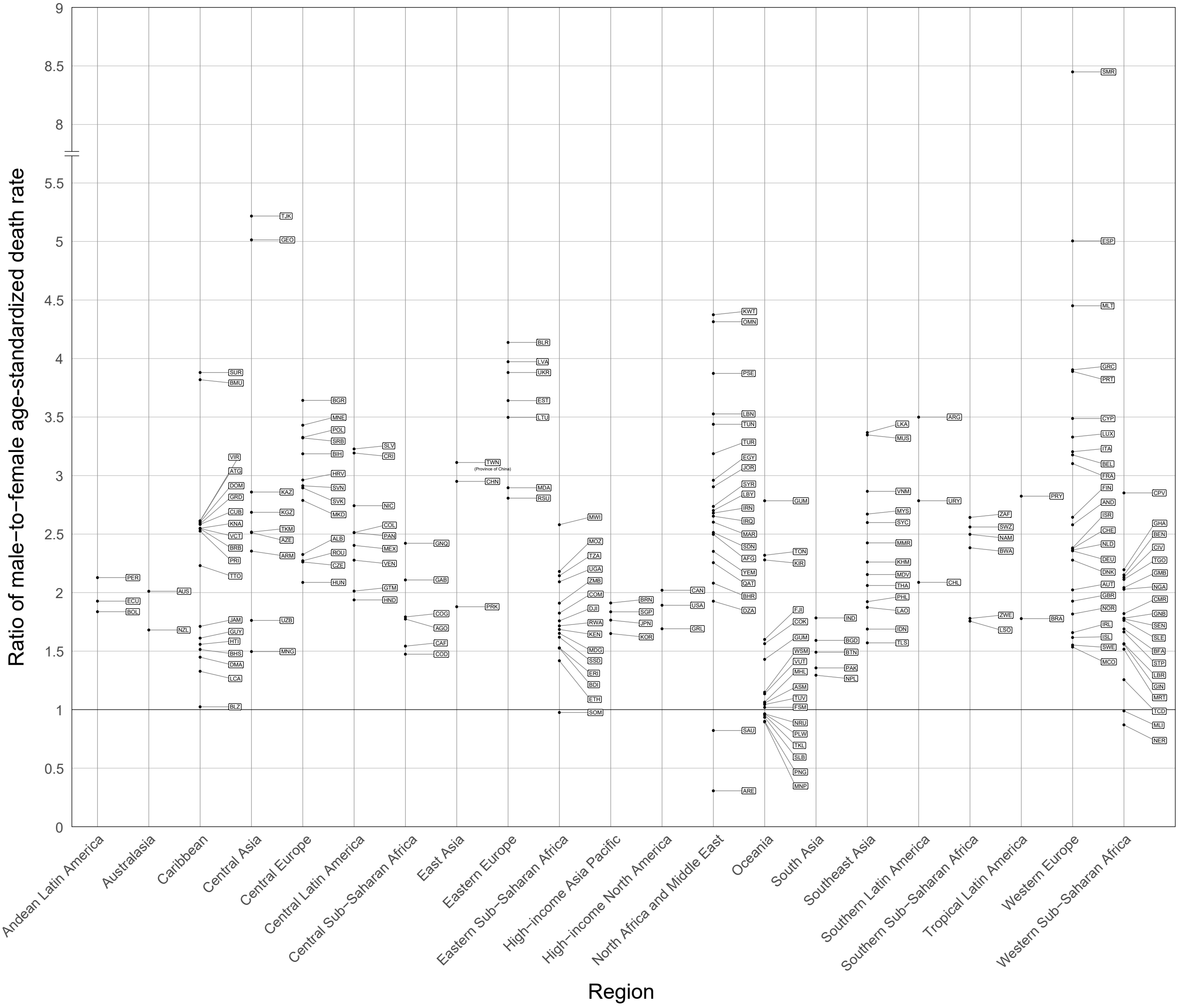

Supplemental Material: Fig. S3

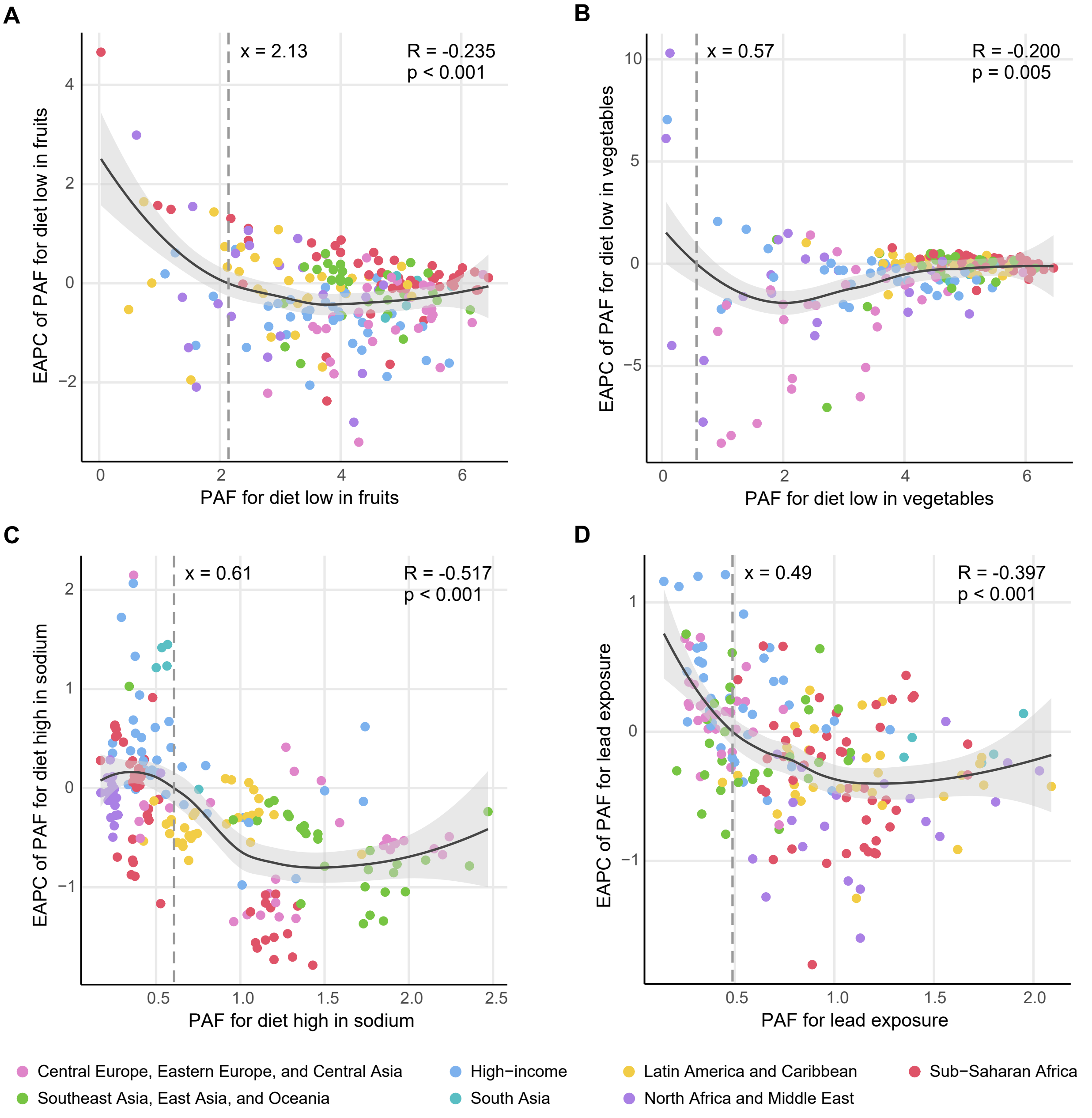

Supplemental Material: Fig. S4

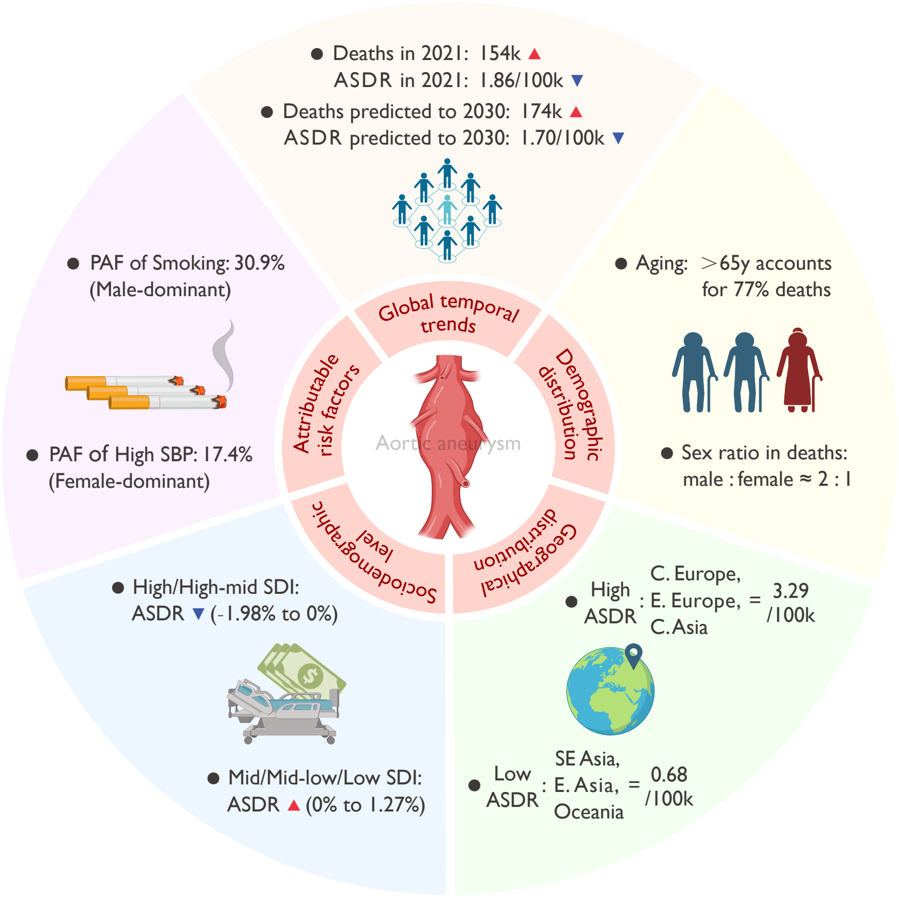

Supplement: Supplementary file 1 — Supplementary file1 (PDF 17041 KB) [file 11739_2025_4061_MOESM1_ESM.pdf]
