# Supplementary material for: Global, regional, and national burden of aortic aneurysm disease and its attributable risk factor, 1990–2021: a systematic analysis for the global burden of disease study 2021
Source: Intern Emerg Med. 2025 Aug 11;20(7):2089–101. doi: 10.1007/s11739-025-04061-8 (PMC12534316; doi:10.1007/s11739-025-04061-8)
Supplement: Supplementary file 2 — Supplementary file2 (DOCX 120 KB) [file 11739_2025_4061_MOESM2_ESM.docx]

**Internal and Emergency Medicine**

**Global, Regional, and National Burden of Aortic Aneurysm Disease and Its Attributable Risk Factor, 1990–2021: A Systematic Analysis for the Global Burden of Disease Study 2021**

**Supplemental Material：Supplemental Tables**

Yue Zhuo^1,#^, Danni Zhao^2,#^, Mingyao Luo^1,3,4,*^, Zhou Zhou^2,**^, Chang Shu^1,***^

Chang Shu, email address: [changshu@vip.126.com](mailto:changshu@vip.126.com); address: No.167, Beilishi Road, Xicheng District, Beijing, China.

| **1** | **Table S1** Global death number, age-standardized death rate, and EAPC in age-standardized death rate for aortic aneurysm by age and sex in 2021 |
| --- | --- |
| **2** | **Table S2** Global age-standardized death rate of aortic aneurysm from 1990 to 2021 |
| **3** | **Table S3** The death number, age-standardized death rate, and EAPC in age-standardized death rate for aortic aneurysm across 204 countries and territories in 2021 |
| **4** | **Table S4** The sex (male to female) ratio of age-standardized death rate for aortic aneurysm across 204 countries and territories in 2021 |
| **5** | **Table S5** The socio-demographic index across 204 countries and territories in 2021 |
| **6** | **Table S6** Global number of population and EAPC in number of populations by age from 1990 to 2021 |
| **7** | **Table S7** The population attributable fraction of risk factors for major vascular disease from 1990 to 2021 |

**Table S1** Global death number, age-standardized death rate, and EAPC in age-standardized death rate for aortic aneurysm by age and sex in 2021

| **Age group** | **1990** |  | **2021** |  | **1990-2021** |
| --- | --- | --- | --- | --- | --- |
|  | **Number (95% UI)** | **Age-standardized rate  (per 100,000) (95% UI)** | **Number (95% UI)** | **Age-standardized rate (per 100,000) (95% UI)** | **EAPC (95% CI)** |
| Both |  |  |  |  |  |
| All ages | 88353 (83090 to 93492) | 2.54 (2.35 to 2.69) | 153927 (138413 to 165739) | 1.86 (1.67 to 2) | -1.28 (-1.38 to -1.18) |
| 15-19 years | 186 (159 to 224) | 0.04 (0.03 to 0.04) | 204 (173 to 239) | 0.03 (0.03 to 0.04) | -0.53 (-0.63 to -0.44) |
| 20-24 years | 305 (263 to 363) | 0.06 (0.05 to 0.07) | 372 (324 to 427) | 0.06 (0.05 to 0.07) | -0.22 (-0.34 to -0.11) |
| 25-29 years | 445 (396 to 519) | 0.1 (0.09 to 0.12) | 589 (521 to 667) | 0.1 (0.09 to 0.11) | -0.05 (-0.1 to 0.02) |
| 30-34 years | 646 (590 to 732) | 0.17 (0.15 to 0.19) | 1015 (912 to 1150) | 0.17 (0.15 to 0.19) | -0.07 (-0.15 to 0.01) |
| 35-39 years | 991 (908 to 1106) | 0.28 (0.26 to 0.31) | 1664 (1485 to 1893) | 0.3 (0.26 to 0.34) | -0.1 (-0.23 to 0.02) |
| 40-44 years | 1338 (1233 to 1493) | 0.47 (0.43 to 0.52) | 2500 (2253 to 2793) | 0.5 (0.45 to 0.56) | -0.16 (-0.29 to -0.03) |
| 45-49 years | 1840 (1710 to 2035) | 0.79 (0.74 to 0.88) | 3728 (3421 to 4118) | 0.79 (0.72 to 0.87) | -0.31 (-0.46 to -0.17) |
| 50-54 years | 2996 (2819 to 3261) | 1.41 (1.33 to 1.53) | 5405 (4984 to 5935) | 1.21 (1.12 to 1.33) | -0.7 (-0.82 to -0.58) |
| 55-59 years | 4666 (4398 to 5075) | 2.52 (2.37 to 2.74) | 8290 (7598 to 9114) | 2.09 (1.92 to 2.3) | -0.81 (-0.94 to -0.68) |
| 60-64 years | 7784 (7392 to 8363) | 4.85 (4.6 to 5.21) | 11669 (10886 to 12673) | 3.65 (3.4 to 3.96) | -1.21 (-1.3 to -1.11) |
| 65-69 years | 11493 (10986 to 12198) | 9.3 (8.89 to 9.87) | 16484 (15355 to 17910) | 5.98 (5.57 to 6.49) | -1.69 (-1.82 to -1.56) |
| 70-74 years | 13329 (12751 to 14068) | 15.74 (15.06 to 16.62) | 21017 (19538 to 22682) | 10.21 (9.49 to 11.02) | -1.95 (-2.12 to -1.79) |
| 75-79 years | 16612 (15714 to 17398) | 26.99 (25.53 to 28.26) | 21508 (19620 to 23035) | 16.31 (14.88 to 17.47) | -1.95 (-2.09 to -1.81) |
| 80-84 years | 13469 (12237 to 14223) | 38.08 (34.59 to 40.2) | 22557 (19647 to 24401) | 25.75 (22.43 to 27.86) | -1.6 (-1.73 to -1.46) |
| 85-89 years | 8192 (7100 to 8742) | 54.21 (46.98 to 57.85) | 19701 (16222 to 21637) | 43.09 (35.48 to 47.32) | -0.88 (-1.03 to -0.73) |
| 90-94 years | 3188 (2629 to 3462) | 74.39 (61.36 to 80.78) | 12188 (9386 to 13647) | 68.13 (52.47 to 76.29) | -0.3 (-0.4 to -0.2) |
| 95+ years | 875 (665 to 976) | 85.91 (65.28 to 95.89) | 5034 (3521 to 5857) | 92.37 (64.6 to 107.47) | 0.22 (0.15 to 0.29) |
| Male |  |  |  |  |  |
| All ages | 57557 (53978 to 62641) | 3.87 (3.61 to 4.18) | 93864 (86610 to 102153) | 2.57 (2.36 to 2.79) | -1.63 (-1.74 to -1.52) |
| 15-19 years | 123 (105 to 156) | 0.05 (0.04 to 0.06) | 131 (108 to 158) | 0.04 (0.03 to 0.05) | -0.67 (-0.77 to -0.58) |
| 20-24 years | 193 (163 to 237) | 0.08 (0.07 to 0.1) | 240 (203 to 286) | 0.08 (0.07 to 0.09) | -0.22 (-0.36 to -0.09) |
| 25-29 years | 293 (250 to 350) | 0.13 (0.11 to 0.16) | 405 (356 to 470) | 0.14 (0.12 to 0.16) | 0.11 (0.04 to 0.17) |
| 30-34 years | 449 (384 to 540) | 0.23 (0.2 to 0.28) | 759 (665 to 882) | 0.25 (0.22 to 0.29) | 0.17 (0.09 to 0.26) |
| 35-39 years | 711 (612 to 849) | 0.4 (0.34 to 0.48) | 1260 (1101 to 1470) | 0.45 (0.39 to 0.52) | 0.12 (0 to 0.24) |
| 40-44 years | 983 (867 to 1170) | 0.67 (0.59 to 0.8) | 1923 (1713 to 2189) | 0.76 (0.68 to 0.87) | 0.05 (-0.07 to 0.18) |
| 45-49 years | 1333 (1182 to 1563) | 1.13 (1 to 1.32) | 2802 (2553 to 3128) | 1.18 (1.07 to 1.32) | -0.11 (-0.26 to 0.05) |
| 50-54 years | 2190 (1991 to 2495) | 2.03 (1.85 to 2.32) | 4021 (3679 to 4456) | 1.81 (1.66 to 2.01) | -0.57 (-0.7 to -0.45) |
| 55-59 years | 3458 (3139 to 3926) | 3.72 (3.38 to 4.23) | 6002 (5485 to 6617) | 3.08 (2.82 to 3.4) | -0.8 (-0.94 to -0.66) |
| 60-64 years | 5828 (5410 to 6464) | 7.42 (6.89 to 8.23) | 8346 (7788 to 9181) | 5.37 (5.01 to 5.9) | -1.32 (-1.42 to -1.22) |
| 65-69 years | 8356 (7809 to 9116) | 14.58 (13.62 to 15.9) | 11426 (10604 to 12616) | 8.67 (8.04 to 9.57) | -1.95 (-2.09 to -1.8) |
| 70-74 years | 9219 (8677 to 9939) | 24.51 (23.06 to 26.42) | 13805 (12950 to 15214) | 14.32 (13.43 to 15.78) | -2.35 (-2.52 to -2.17) |
| 75-79 years | 10764 (10174 to 11463) | 42.66 (40.32 to 45.43) | 13297 (12314 to 14558) | 22.24 (20.6 to 24.35) | -2.43 (-2.58 to -2.28) |
| 80-84 years | 7914 (7307 to 8406) | 59.58 (55.01 to 63.28) | 12727 (11357 to 13895) | 34.72 (30.99 to 37.91) | -2.1 (-2.25 to -1.95) |
| 85-89 years | 4111 (3686 to 4397) | 81.17 (72.79 to 86.82) | 9997 (8696 to 10773) | 57.94 (50.41 to 62.44) | -1.29 (-1.46 to -1.12) |
| 90-94 years | 1339 (1161 to 1445) | 106.36 (92.21 to 114.77) | 5204 (4387 to 5674) | 89.29 (75.27 to 97.35) | -0.68 (-0.81 to -0.55) |
| 95+ years | 293 (236 to 322) | 112.55 (90.71 to 123.55) | 1520 (1157 to 1697) | 100.5 (76.52 to 112.24) | -0.59 (-0.67 to -0.5) |
| Female |  |  |  |  |  |
| All ages | 30796 (27622 to 34388) | 1.58 (1.41 to 1.76) | 60063 (51303 to 66298) | 1.28 (1.1 to 1.42) | -0.91 (-1.01 to -0.81) |
| 15-19 years | 62 (48 to 92) | 0.02 (0.02 to 0.04) | 73 (60 to 88) | 0.02 (0.02 to 0.03) | -0.31 (-0.42 to -0.2) |
| 20-24 years | 111 (85 to 163) | 0.05 (0.03 to 0.07) | 133 (110 to 163) | 0.05 (0.04 to 0.06) | -0.24 (-0.35 to -0.14) |
| 25-29 years | 152 (120 to 215) | 0.07 (0.05 to 0.1) | 184 (153 to 218) | 0.06 (0.05 to 0.07) | -0.36 (-0.43 to -0.28) |
| 30-34 years | 196 (161 to 268) | 0.1 (0.08 to 0.14) | 256 (227 to 292) | 0.09 (0.08 to 0.1) | -0.67 (-0.73 to -0.61) |
| 35-39 years | 280 (234 to 367) | 0.16 (0.13 to 0.21) | 404 (364 to 444) | 0.15 (0.13 to 0.16) | -0.67 (-0.82 to -0.53) |
| 40-44 years | 355 (306 to 449) | 0.25 (0.22 to 0.32) | 577 (515 to 638) | 0.23 (0.21 to 0.26) | -0.74 (-0.9 to -0.58) |
| 45-49 years | 507 (437 to 641) | 0.45 (0.38 to 0.56) | 925 (828 to 1026) | 0.39 (0.35 to 0.44) | -0.8 (-0.94 to -0.67) |
| 50-54 years | 805 (706 to 990) | 0.77 (0.67 to 0.94) | 1384 (1250 to 1553) | 0.62 (0.56 to 0.7) | -0.92 (-1.04 to -0.81) |
| 55-59 years | 1208 (1067 to 1469) | 1.31 (1.16 to 1.59) | 2288 (2040 to 2587) | 1.14 (1.01 to 1.29) | -0.72 (-0.85 to -0.59) |
| 60-64 years | 1956 (1761 to 2293) | 2.38 (2.15 to 2.79) | 3323 (3007 to 3779) | 2.02 (1.83 to 2.3) | -0.84 (-0.94 to -0.75) |
| 65-69 years | 3137 (2872 to 3545) | 4.73 (4.33 to 5.35) | 5058 (4610 to 5562) | 3.51 (3.2 to 3.86) | -1.17 (-1.28 to -1.06) |
| 70-74 years | 4110 (3762 to 4605) | 8.74 (8 to 9.79) | 7212 (6484 to 8060) | 6.59 (5.92 to 7.36) | -1.38 (-1.52 to -1.23) |
| 75-79 years | 5848 (5360 to 6369) | 16.1 (14.76 to 17.54) | 8211 (7266 to 8967) | 11.39 (10.08 to 12.44) | -1.43 (-1.57 to -1.29) |
| 80-84 years | 5556 (4882 to 6005) | 25.15 (22.1 to 27.18) | 9830 (8218 to 10872) | 19.3 (16.14 to 21.35) | -1.19 (-1.33 to -1.05) |
| 85-89 years | 4081 (3399 to 4448) | 40.62 (33.83 to 44.27) | 9705 (7419 to 11081) | 34.09 (26.06 to 38.92) | -0.66 (-0.81 to -0.51) |
| 90-94 years | 1849 (1461 to 2037) | 61.09 (48.27 to 67.31) | 6984 (5046 to 8073) | 57.91 (41.84 to 66.94) | -0.12 (-0.21 to -0.02) |
| 95+ years | 582 (433 to 657) | 76.76 (57.19 to 86.73) | 3515 (2367 to 4171) | 89.24 (60.11 to 105.9) | 0.56 (0.47 to 0.65) |

UI, uncertainty intervals; EAPC, estimated annual percentage change; CI, confidence interval

**Table S2** Global age-standardized death rate of aortic aneurysm from 1990 to 2021

| **Year** | **Both** | **Male** | **Female** |
| --- | --- | --- | --- |
| 1990 | 2.54 (2.35 to 2.69) | 3.87 (3.61 to 4.18) | 1.58 (1.41 to 1.76) |
| 1991 | 2.56 (2.37 to 2.72) | 3.89 (3.63 to 4.2) | 1.6 (1.43 to 1.77) |
| 1992 | 2.57 (2.37 to 2.72) | 3.9 (3.64 to 4.21) | 1.6 (1.43 to 1.77) |
| 1993 | 2.6 (2.4 to 2.75) | 3.95 (3.69 to 4.25) | 1.64 (1.47 to 1.81) |
| 1994 | 2.61 (2.42 to 2.77) | 3.94 (3.68 to 4.24) | 1.64 (1.47 to 1.81) |
| 1995 | 2.62 (2.42 to 2.77) | 3.94 (3.68 to 4.24) | 1.61 (1.43 to 1.78) |
| 1996 | 2.59 (2.4 to 2.74) | 3.89 (3.64 to 4.19) | 1.61 (1.43 to 1.77) |
| 1997 | 2.55 (2.35 to 2.7) | 3.81 (3.57 to 4.11) | 1.65 (1.46 to 1.81) |
| 1998 | 2.53 (2.33 to 2.68) | 3.76 (3.52 to 4.06) | 1.63 (1.44 to 1.78) |
| 1999 | 2.54 (2.35 to 2.7) | 3.74 (3.5 to 4.02) | 1.57 (1.38 to 1.72) |
| 2000 | 2.5 (2.31 to 2.65) | 3.67 (3.42 to 3.95) | 1.53 (1.34 to 1.67) |
| 2001 | 2.42 (2.22 to 2.57) | 3.56 (3.32 to 3.83) | 1.51 (1.32 to 1.65) |
| 2002 | 2.39 (2.19 to 2.54) | 3.52 (3.28 to 3.79) | 1.49 (1.3 to 1.63) |
| 2003 | 2.36 (2.16 to 2.51) | 3.46 (3.23 to 3.73) | 1.46 (1.27 to 1.6) |
| 2004 | 2.31 (2.11 to 2.46) | 3.37 (3.14 to 3.64) | 1.44 (1.25 to 1.58) |
| 2005 | 2.28 (2.08 to 2.43) | 3.32 (3.09 to 3.58) | 1.41 (1.23 to 1.55) |
| 2006 | 2.22 (2.02 to 2.36) | 3.23 (3 to 3.48) | 1.39 (1.21 to 1.53) |
| 2007 | 2.19 (1.99 to 2.33) | 3.16 (2.94 to 3.41) | 1.37 (1.19 to 1.51) |
| 2008 | 2.14 (1.94 to 2.28) | 3.09 (2.87 to 3.34) | 1.36 (1.17 to 1.49) |
| 2009 | 2.1 (1.91 to 2.24) | 3 (2.79 to 3.25) | 1.34 (1.16 to 1.47) |
| 2010 | 2.07 (1.88 to 2.2) | 2.95 (2.73 to 3.19) | 1.33 (1.15 to 1.46) |
| 2011 | 2.02 (1.84 to 2.16) | 2.87 (2.66 to 3.1) | 1.33 (1.15 to 1.46) |
| 2012 | 1.99 (1.8 to 2.13) | 2.8 (2.59 to 3.03) | 1.33 (1.15 to 1.46) |
| 2013 | 1.96 (1.78 to 2.11) | 2.75 (2.53 to 3) | 1.34 (1.15 to 1.46) |
| 2014 | 1.95 (1.76 to 2.1) | 2.72 (2.52 to 2.97) | 1.33 (1.14 to 1.45) |
| 2015 | 1.94 (1.76 to 2.09) | 2.71 (2.49 to 2.93) | 1.32 (1.13 to 1.44) |
| 2016 | 1.95 (1.75 to 2.08) | 2.7 (2.48 to 2.92) | 1.31 (1.13 to 1.43) |
| 2017 | 1.92 (1.74 to 2.07) | 2.66 (2.46 to 2.88) | 1.28 (1.1 to 1.42) |
| 2018 | 1.91 (1.72 to 2.05) | 2.64 (2.44 to 2.85) | 1.64 (1.45 to 1.8) |
| 2019 | 1.9 (1.71 to 2.04) | 2.62 (2.41 to 2.83) | 1.54 (1.36 to 1.69) |
| 2020 | 1.87 (1.67 to 2) | 2.58 (2.37 to 2.8) | 1.62 (1.45 to 1.79) |
| 2021 | 1.86 (1.67 to 2) | 2.57 (2.36 to 2.79) | 1.29 (1.09 to 1.41) |

UI, uncertainty intervals

**Table S3** The death number, age-standardized death rate, and EAPC in age-standardized death rate for aortic aneurysm across 204 countries and territories in 2021

| **Location** | **1990** |  | **2021** |  | **1990-2021** |
| --- | --- | --- | --- | --- | --- |
|  | **Number (95% UI)** | **Age-standardized rate (per 100,000) (95% UI)** | **Number (95% UI)** | **Age-standardized rate (per 100,000) (95% UI)** | **EAPC (95% CI)** |
| Central Europe, Eastern Europe, and Central Asia |  |  |  |  |  |
| Central Asia |  |  |  |  |  |
| Armenia | 117 (96 to 142) | 4.55 (3.71 to 5.57) | 397 (330 to 467) | 9.16 (7.61 to 10.81) | 2.57 (2.21 to 2.94) |
| Azerbaijan | 35 (26 to 46) | 0.73 (0.55 to 0.96) | 130 (81 to 212) | 1.37 (0.93 to 2.08) | 2.58 (2.27 to 2.9) |
| Georgia | 37 (31 to 44) | 0.61 (0.51 to 0.72) | 173 (146 to 200) | 2.88 (2.43 to 3.35) | 7.38 (6.07 to 8.7) |
| Kazakhstan | 180 (147 to 231) | 1.46 (1.19 to 1.87) | 377 (302 to 462) | 2.23 (1.8 to 2.71) | 0.55 (0.18 to 0.91) |
| Kyrgyzstan | 8 (7 to 9) | 0.28 (0.24 to 0.33) | 36 (29 to 44) | 0.79 (0.63 to 0.96) | 4.03 (3.43 to 4.64) |
| Mongolia | 3 (2 to 4) | 0.31 (0.22 to 0.42) | 14 (11 to 18) | 0.7 (0.53 to 0.89) | 2.88 (2.52 to 3.24) |
| Tajikistan | 7 (5 to 9) | 0.28 (0.19 to 0.36) | 13 (9 to 17) | 0.25 (0.18 to 0.34) | -0.45 (-0.7 to -0.19) |
| Turkmenistan | 19 (15 to 23) | 1.03 (0.84 to 1.26) | 72 (53 to 103) | 1.88 (1.42 to 2.67) | 1.83 (1.66 to 2) |
| Uzbekistan | 24 (18 to 34) | 0.21 (0.16 to 0.3) | 231 (186 to 285) | 1.04 (0.85 to 1.28) | 5.79 (4.99 to 6.59) |
| Central Europe |  |  |  |  |  |
| Albania | 17 (14 to 20) | 0.91 (0.76 to 1.08) | 47 (32 to 65) | 1.09 (0.76 to 1.5) | 0.73 (0.59 to 0.86) |
| Bosnia and Herzegovina | 79 (58 to 109) | 2.12 (1.55 to 2.89) | 189 (135 to 252) | 3 (2.15 to 3.99) | 1.31 (1.18 to 1.43) |
| Bulgaria | 217 (193 to 240) | 2.03 (1.82 to 2.23) | 363 (295 to 449) | 2.62 (2.12 to 3.23) | 0.74 (0.46 to 1.02) |
| Croatia | 157 (137 to 180) | 2.79 (2.45 to 3.19) | 317 (271 to 365) | 3.34 (2.87 to 3.86) | 0.48 (0.23 to 0.72) |
| Czechia | 405 (377 to 436) | 2.96 (2.76 to 3.18) | 637 (554 to 722) | 2.83 (2.46 to 3.19) | -0.24 (-0.66 to 0.18) |
| Hungary | 460 (426 to 496) | 3.23 (3 to 3.48) | 574 (507 to 647) | 2.87 (2.54 to 3.23) | -0.71 (-0.86 to -0.56) |
| Montenegro | 40 (32 to 52) | 6.7 (5.35 to 8.64) | 81 (61 to 105) | 8.65 (6.59 to 11.28) | 1.11 (0.93 to 1.29) |
| North Macedonia | 33 (26 to 41) | 1.94 (1.51 to 2.42) | 80 (51 to 120) | 2.63 (1.8 to 3.81) | 0.72 (0.46 to 0.97) |
| Poland | 1943 (1876 to 1997) | 4.54 (4.36 to 4.66) | 2576 (2307 to 2831) | 3.44 (3.09 to 3.79) | -1.35 (-1.57 to -1.13) |
| Romania | 360 (334 to 391) | 1.4 (1.3 to 1.52) | 706 (614 to 810) | 1.9 (1.65 to 2.18) | 0.69 (0.53 to 0.85) |
| Serbia | 397 (330 to 474) | 4.22 (3.5 to 5.14) | 692 (549 to 872) | 4.05 (3.21 to 5.1) | -0.07 (-0.15 to 0.01) |
| Slovakia | 135 (116 to 158) | 2.29 (1.97 to 2.68) | 218 (170 to 275) | 2.29 (1.79 to 2.9) | 0.01 (-0.1 to 0.12) |
| Slovenia | 65 (61 to 70) | 2.66 (2.47 to 2.84) | 105 (89 to 126) | 2.16 (1.82 to 2.58) | -0.94 (-1.27 to -0.62) |
| Eastern Europe |  |  |  |  |  |
| Belarus | 338 (294 to 411) | 2.67 (2.31 to 3.23) | 596 (486 to 715) | 3.75 (3.05 to 4.49) | 0.72 (0.37 to 1.08) |
| Estonia | 58 (52 to 63) | 2.86 (2.6 to 3.13) | 98 (85 to 110) | 3.34 (2.9 to 3.78) | 0.03 (-0.32 to 0.38) |
| Latvia | 79 (72 to 86) | 2.21 (2.03 to 2.4) | 125 (107 to 142) | 3.05 (2.63 to 3.47) | 0.54 (0.21 to 0.87) |
| Lithuania | 97 (88 to 106) | 2.16 (1.97 to 2.36) | 187 (162 to 211) | 3.15 (2.73 to 3.56) | 1.05 (0.82 to 1.29) |
| Republic of Moldova | 39 (35 to 43) | 0.96 (0.89 to 1.06) | 89 (79 to 100) | 1.49 (1.32 to 1.67) | 1.26 (0.87 to 1.64) |
| Russian Federation | 4699 (4559 to 4800) | 2.72 (2.62 to 2.78) | 10445 (9555 to 11307) | 4.38 (4.01 to 4.74) | 1.22 (0.75 to 1.69) |
| Ukraine | 1503 (1356 to 1672) | 2.13 (1.92 to 2.37) | 1867 (1385 to 2442) | 2.47 (1.82 to 3.25) | 0.06 (-0.25 to 0.38) |
| High-income |  |  |  |  |  |
| Australasia |  |  |  |  |  |
| Australia | 1490 (1385 to 1588) | 7.57 (7.02 to 8.06) | 1186 (1033 to 1292) | 2.35 (2.06 to 2.55) | -4.27 (-4.43 to -4.1) |
| New Zealand | 412 (382 to 438) | 10.27 (9.5 to 10.91) | 362 (318 to 392) | 3.99 (3.52 to 4.3) | -3.52 (-3.69 to -3.35) |
| High-income Asia Pacific |  |  |  |  |  |
| Brunei Darussalam | 5 (4 to 6) | 5.61 (4.47 to 6.89) | 13 (11 to 15) | 4.79 (4 to 5.67) | -0.03 (-0.21 to 0.14) |
| Japan | 4797 (4448 to 4982) | 2.92 (2.68 to 3.04) | 23815 (19180 to 26463) | 5.07 (4.33 to 5.47) | 1.92 (1.78 to 2.06) |
| Republic of Korea | 422 (280 to 603) | 1.84 (1.25 to 2.57) | 1767 (1441 to 2093) | 1.92 (1.56 to 2.27) | 0.07 (-0.15 to 0.28) |
| Singapore | 53 (50 to 55) | 2.65 (2.49 to 2.79) | 177 (158 to 191) | 2.15 (1.91 to 2.32) | -0.55 (-0.75 to -0.35) |
| High-income North America |  |  |  |  |  |
| Canada | 2168 (2000 to 2353) | 6.6 (6.08 to 7.13) | 1774 (1541 to 1929) | 2.26 (1.99 to 2.45) | -4.1 (-4.36 to -3.83) |
| Greenland | 1 (1 to 1) | 4.05 (3.57 to 4.59) | 1 (1 to 1) | 1.84 (1.49 to 2.3) | -2.71 (-2.87 to -2.55) |
| United States of America | 17399 (16107 to 18106) | 5.17 (4.8 to 5.38) | 12195 (10940 to 12944) | 2.06 (1.87 to 2.18) | -3.61 (-3.86 to -3.36) |
| Southern Latin America |  |  |  |  |  |
| Argentina | 1637 (1508 to 1798) | 5.19 (4.78 to 5.68) | 1533 (1398 to 1665) | 2.67 (2.44 to 2.9) | -2.24 (-2.44 to -2.03) |
| Chile | 256 (241 to 268) | 2.7 (2.56 to 2.84) | 563 (514 to 604) | 2.17 (1.98 to 2.32) | -0.79 (-1.1 to -0.48) |
| Uruguay | 228 (213 to 247) | 5.81 (5.42 to 6.24) | 256 (233 to 276) | 4.35 (3.98 to 4.67) | -1.3 (-1.54 to -1.06) |
| Western Europe |  |  |  |  |  |
| Andorra | 4 (2 to 5) | 6.87 (4.36 to 10.33) | 6 (4 to 10) | 3.96 (2.5 to 5.97) | -1.72 (-1.93 to -1.5) |
| Austria | 397 (373 to 419) | 3.2 (3.03 to 3.37) | 365 (321 to 395) | 1.8 (1.61 to 1.94) | -2.11 (-2.28 to -1.93) |
| Belgium | 821 (759 to 879) | 5.11 (4.73 to 5.45) | 606 (517 to 659) | 2.25 (1.96 to 2.43) | -2.92 (-3.1 to -2.73) |
| Cyprus | 50 (39 to 64) | 7.82 (6.01 to 10.06) | 80 (60 to 101) | 3.96 (3.04 to 4.88) | -2.66 (-2.87 to -2.44) |
| Denmark | 575 (530 to 616) | 6.71 (6.21 to 7.17) | 617 (547 to 673) | 4.61 (4.1 to 5.02) | -1.72 (-2.06 to -1.38) |
| Finland | 578 (538 to 614) | 7.88 (7.35 to 8.36) | 519 (450 to 566) | 3.63 (3.2 to 3.93) | -2.46 (-2.7 to -2.23) |
| France | 3206 (2980 to 3386) | 3.65 (3.41 to 3.85) | 3145 (2713 to 3401) | 1.89 (1.67 to 2.04) | -2.63 (-2.91 to -2.35) |
| Germany | 4459 (4073 to 4774) | 3.32 (3.07 to 3.56) | 4735 (4139 to 5131) | 2.25 (2.02 to 2.41) | -1.46 (-1.59 to -1.33) |
| Greece | 574 (536 to 611) | 3.81 (3.55 to 4.03) | 1066 (955 to 1149) | 3.99 (3.65 to 4.26) | -0.41 (-0.71 to -0.11) |
| Iceland | 13 (12 to 14) | 4.34 (3.91 to 4.65) | 17 (14 to 19) | 2.61 (2.23 to 2.92) | -2.02 (-2.34 to -1.7) |
| Ireland | 266 (249 to 284) | 6.36 (5.94 to 6.78) | 248 (207 to 275) | 2.93 (2.47 to 3.25) | -2.85 (-3.18 to -2.51) |
| Israel | 112 (103 to 120) | 2.34 (2.15 to 2.5) | 171 (150 to 187) | 1.28 (1.13 to 1.4) | -2.31 (-2.51 to -2.11) |
| Italy | 2816 (2646 to 2922) | 3.1 (2.91 to 3.21) | 3655 (3187 to 3939) | 2.18 (1.95 to 2.34) | -1.74 (-2.07 to -1.42) |
| Luxembourg | 24 (23 to 26) | 4.33 (4.06 to 4.64) | 26 (22 to 28) | 2.23 (1.97 to 2.46) | -2.67 (-2.9 to -2.43) |
| Malta | 11 (10 to 12) | 2.6 (2.41 to 2.8) | 14 (12 to 16) | 1.32 (1.17 to 1.47) | -2.76 (-3.12 to -2.4) |
| Monaco | 4 (3 to 6) | 5.66 (4.29 to 7.02) | 6 (4 to 8) | 5.15 (3.88 to 6.95) | -0.28 (-0.45 to -0.11) |
| Netherlands | 1550 (1430 to 1657) | 7.44 (6.89 to 7.96) | 1466 (1263 to 1605) | 3.74 (3.24 to 4.08) | -2.83 (-3.19 to -2.46) |
| Norway | 573 (539 to 598) | 7.58 (7.16 to 7.9) | 541 (466 to 581) | 4.75 (4.15 to 5.08) | -2.08 (-2.39 to -1.77) |
| Portugal | 238 (223 to 253) | 1.75 (1.64 to 1.86) | 405 (361 to 437) | 1.51 (1.37 to 1.62) | -0.86 (-1.06 to -0.67) |
| San Marino | 1 (1 to 1) | 3.09 (2.49 to 3.76) | 1 (1 to 2) | 1.67 (1.06 to 2.59) | -0.89 (-1.29 to -0.49) |
| Spain | 1402 (1313 to 1480) | 2.54 (2.37 to 2.68) | 2256 (1979 to 2434) | 2.08 (1.87 to 2.23) | -1.3 (-1.61 to -1) |
| Sweden | 1226 (1141 to 1295) | 7.41 (6.91 to 7.79) | 985 (846 to 1098) | 3.88 (3.38 to 4.33) | -2.53 (-2.84 to -2.22) |
| Switzerland | 541 (487 to 585) | 4.87 (4.43 to 5.26) | 488 (411 to 540) | 2.28 (1.96 to 2.51) | -2.51 (-2.68 to -2.33) |
| United Kingdom | 9552 (9013 to 9824) | 9.72 (9.16 to 10) | 6071 (5374 to 6435) | 4.03 (3.61 to 4.25) | -3.41 (-3.78 to -3.05) |
| Latin America and Caribbean |  |  |  |  |  |
| Andean Latin America |  |  |  |  |  |
| Bolivia (Plurinational State of) | 35 (24 to 51) | 1.24 (0.84 to 1.79) | 111 (84 to 150) | 1.36 (1.03 to 1.84) | 0.36 (0.32 to 0.4) |
| Ecuador | 69 (64 to 74) | 1.39 (1.29 to 1.49) | 208 (165 to 262) | 1.32 (1.06 to 1.66) | 0.35 (-0.03 to 0.73) |
| Peru | 89 (69 to 114) | 0.78 (0.61 to 1.02) | 219 (163 to 290) | 0.66 (0.49 to 0.87) | -0.66 (-0.81 to -0.51) |
| Caribbean |  |  |  |  |  |
| Antigua and Barbuda | 2 (2 to 2) | 2.95 (2.69 to 3.26) | 2 (2 to 2) | 1.92 (1.75 to 2.15) | -1.95 (-2.24 to -1.66) |
| Bahamas | 5 (5 to 6) | 3.75 (3.39 to 4.22) | 10 (9 to 12) | 2.85 (2.37 to 3.4) | -1.09 (-1.44 to -0.74) |
| Barbados | 9 (8 to 10) | 2.88 (2.67 to 3.12) | 12 (9 to 14) | 2.18 (1.77 to 2.68) | -1.54 (-1.85 to -1.23) |
| Belize | 1 (1 to 1) | 1 (0.88 to 1.17) | 2 (2 to 3) | 0.88 (0.76 to 0.99) | -1.1 (-1.81 to -0.39) |
| Bermuda | 7 (6 to 7) | 11.69 (10.86 to 12.53) | 6 (5 to 8) | 4.15 (3.55 to 4.99) | -3.48 (-3.63 to -3.33) |
| Cuba | 572 (525 to 617) | 5.72 (5.25 to 6.15) | 770 (655 to 868) | 3.74 (3.18 to 4.22) | -1.76 (-1.96 to -1.56) |
| Dominica | 2 (2 to 3) | 3.85 (2.97 to 4.81) | 3 (2 to 4) | 3.94 (3.17 to 4.87) | -0.26 (-0.46 to -0.06) |
| Dominican Republic | 46 (38 to 57) | 1.46 (1.2 to 1.78) | 158 (115 to 208) | 1.63 (1.19 to 2.15) | 0.27 (0.08 to 0.46) |
| Grenada | 4 (3 to 4) | 4.77 (4.15 to 5.7) | 5 (4 to 5) | 4.51 (3.94 to 5.04) | -1.02 (-1.74 to -0.28) |
| Guyana | 7 (6 to 7) | 1.99 (1.79 to 2.19) | 20 (15 to 25) | 3.45 (2.7 to 4.33) | 1.28 (0.57 to 1.99) |
| Haiti | 60 (39 to 93) | 2.36 (1.56 to 3.53) | 131 (81 to 205) | 2.29 (1.42 to 3.57) | -0.1 (-0.14 to -0.05) |
| Jamaica | 33 (29 to 37) | 1.75 (1.58 to 1.96) | 56 (43 to 70) | 1.75 (1.36 to 2.21) | -0.18 (-0.5 to 0.13) |
| Puerto Rico | 81 (76 to 86) | 2.34 (2.18 to 2.47) | 77 (63 to 90) | 0.93 (0.77 to 1.08) | -3.69 (-3.93 to -3.44) |
| Saint Kitts and Nevis | 1 (1 to 2) | 3.69 (3.35 to 4.05) | 1 (1 to 2) | 2.44 (2.04 to 2.9) | -1.58 (-1.82 to -1.35) |
| Saint Lucia | 8 (7 to 9) | 10.31 (9.41 to 11.56) | 11 (10 to 13) | 4.95 (4.17 to 5.77) | -3.29 (-3.64 to -2.93) |
| Saint Vincent and the Grenadines | 2 (2 to 2) | 2.56 (2.36 to 2.77) | 3 (2 to 3) | 2.09 (1.85 to 2.35) | -1.22 (-1.54 to -0.9) |
| Suriname | 6 (5 to 7) | 2.39 (2.01 to 2.83) | 12 (8 to 17) | 2.02 (1.41 to 2.79) | -0.59 (-0.75 to -0.43) |
| Trinidad and Tobago | 41 (38 to 45) | 5.46 (4.99 to 5.96) | 74 (57 to 94) | 3.94 (3.05 to 4.96) | -1.57 (-1.88 to -1.26) |
| United States Virgin Islands | 2 (2 to 3) | 3.29 (2.72 to 3.93) | 4 (3 to 5) | 2.02 (1.56 to 2.57) | -1.71 (-1.9 to -1.53) |
| Central Latin America |  |  |  |  |  |
| Colombia | 576 (545 to 606) | 3.48 (3.28 to 3.65) | 1636 (1357 to 1937) | 2.97 (2.47 to 3.53) | -1.63 (-2.01 to -1.24) |
| Costa Rica | 39 (36 to 44) | 2.34 (2.13 to 2.6) | 132 (115 to 148) | 2.41 (2.1 to 2.7) | -0.4 (-0.65 to -0.15) |
| El Salvador | 15 (13 to 18) | 0.53 (0.44 to 0.63) | 34 (27 to 43) | 0.53 (0.42 to 0.68) | -0.19 (-0.34 to -0.04) |
| Guatemala | 17 (16 to 19) | 0.6 (0.54 to 0.66) | 45 (38 to 52) | 0.43 (0.37 to 0.5) | -1.68 (-1.94 to -1.41) |
| Honduras | 15 (11 to 20) | 0.8 (0.6 to 1.07) | 66 (48 to 90) | 1.19 (0.87 to 1.6) | 1.41 (1.28 to 1.54) |
| Mexico | 293 (284 to 300) | 0.79 (0.76 to 0.81) | 750 (662 to 845) | 0.63 (0.56 to 0.71) | -1.01 (-1.18 to -0.85) |
| Nicaragua | 5 (4 to 6) | 0.34 (0.29 to 0.4) | 16 (13 to 19) | 0.34 (0.28 to 0.42) | 0.05 (-0.19 to 0.29) |
| Panama | 32 (29 to 34) | 2.24 (2.03 to 2.42) | 79 (60 to 96) | 1.77 (1.35 to 2.15) | -1.22 (-1.43 to -1) |
| Venezuela (Bolivarian Republic of) | 205 (192 to 221) | 2.27 (2.11 to 2.45) | 536 (419 to 671) | 1.91 (1.5 to 2.37) | -1.1 (-1.39 to -0.8) |
| Tropical Latin America |  |  |  |  |  |
| Brazil | 2857 (2739 to 2952) | 3.39 (3.23 to 3.52) | 10010 (9186 to 10566) | 4.06 (3.72 to 4.29) | 0.34 (0.09 to 0.59) |
| Paraguay | 43 (35 to 54) | 2.03 (1.64 to 2.52) | 163 (126 to 207) | 2.9 (2.23 to 3.67) | 1.29 (1.18 to 1.4) |
| North Africa and Middle East |  |  |  |  |  |
| Afghanistan | 6 (3 to 10) | 0.09 (0.05 to 0.15) | 29 (18 to 44) | 0.29 (0.19 to 0.42) | 4.57 (4.28 to 4.87) |
| Algeria | 17 (12 to 23) | 0.16 (0.11 to 0.22) | 128 (96 to 170) | 0.42 (0.32 to 0.55) | 3.92 (3.55 to 4.3) |
| Bahrain | 1 (1 to 1) | 0.63 (0.52 to 0.8) | 5 (4 to 7) | 0.71 (0.56 to 0.9) | 0.53 (0.11 to 0.95) |
| Egypt | 87 (63 to 128) | 0.36 (0.26 to 0.52) | 250 (200 to 308) | 0.45 (0.36 to 0.56) | 0.81 (0.72 to 0.89) |
| Iran (Islamic Republic of) | 65 (53 to 81) | 0.29 (0.23 to 0.35) | 384 (340 to 432) | 0.53 (0.47 to 0.6) | 2.86 (2.47 to 3.25) |
| Iraq | 24 (17 to 33) | 0.29 (0.21 to 0.39) | 89 (65 to 118) | 0.38 (0.28 to 0.48) | 0.72 (0.64 to 0.79) |
| Jordan | 15 (11 to 19) | 1.12 (0.86 to 1.46) | 69 (52 to 89) | 0.97 (0.76 to 1.24) | -0.57 (-0.76 to -0.38) |
| Kuwait | 6 (6 to 7) | 0.97 (0.88 to 1.07) | 24 (20 to 30) | 0.86 (0.71 to 1.06) | 0.12 (-0.82 to 1.07) |
| Lebanon | 59 (30 to 106) | 2.85 (1.41 to 5.16) | 140 (114 to 175) | 2.21 (1.8 to 2.76) | -0.71 (-0.97 to -0.44) |
| Libya | 2 (2 to 4) | 0.12 (0.08 to 0.18) | 18 (11 to 29) | 0.35 (0.21 to 0.54) | 4.23 (3.81 to 4.64) |
| Morocco | 18 (12 to 26) | 0.13 (0.09 to 0.18) | 140 (98 to 182) | 0.44 (0.31 to 0.57) | 4.53 (4.3 to 4.76) |
| Oman | 1 (1 to 2) | 0.17 (0.11 to 0.27) | 11 (7 to 19) | 0.56 (0.35 to 0.88) | 4.64 (4.19 to 5.1) |
| Palestine | 5 (4 to 7) | 0.62 (0.44 to 0.82) | 17 (14 to 22) | 0.77 (0.58 to 0.98) | 0.72 (0.62 to 0.81) |
| Qatar | 1 (1 to 2) | 1.46 (1.18 to 1.82) | 8 (5 to 13) | 0.94 (0.59 to 1.46) | -1.71 (-2.21 to -1.2) |
| Saudi Arabia | 4 (3 to 6) | 0.08 (0.05 to 0.11) | 41 (29 to 55) | 0.21 (0.16 to 0.28) | 3.95 (3.54 to 4.36) |
| Sudan | 10 (5 to 22) | 0.1 (0.06 to 0.23) | 67 (41 to 98) | 0.34 (0.22 to 0.49) | 4.52 (4.26 to 4.78) |
| Syrian Arab Republic | 18 (12 to 25) | 0.35 (0.25 to 0.47) | 51 (37 to 68) | 0.42 (0.31 to 0.56) | 0.52 (0.42 to 0.61) |
| Tunisia | 7 (5 to 10) | 0.15 (0.11 to 0.21) | 56 (36 to 81) | 0.45 (0.29 to 0.66) | 3.95 (3.64 to 4.26) |
| Turkiye | 700 (495 to 995) | 2.05 (1.48 to 2.9) | 2075 (1640 to 2606) | 2.27 (1.8 to 2.83) | 0.35 (0.13 to 0.57) |
| United Arab Emirates | 6 (4 to 9) | 1.27 (0.82 to 1.82) | 43 (34 to 54) | 1.45 (1.17 to 1.84) | 2.47 (1.69 to 3.25) |
| Yemen | 5 (2 to 9) | 0.1 (0.05 to 0.18) | 46 (26 to 71) | 0.33 (0.2 to 0.51) | 4.47 (4.1 to 4.84) |
| South Asia |  |  |  |  |  |
| Bangladesh | 334 (196 to 596) | 0.82 (0.49 to 1.42) | 1458 (952 to 2422) | 1.21 (0.8 to 1.99) | 1.3 (1.14 to 1.46) |
| Bhutan | 1 (1 to 2) | 0.78 (0.51 to 1.16) | 8 (5 to 12) | 1.49 (0.93 to 2.16) | 2.31 (2.25 to 2.38) |
| India | 2652 (1578 to 4344) | 0.68 (0.4 to 1.08) | 12805 (9107 to 18763) | 1.2 (0.86 to 1.74) | 2.07 (1.87 to 2.26) |
| Nepal | 48 (27 to 86) | 0.64 (0.37 to 1.13) | 236 (162 to 368) | 1.19 (0.82 to 1.85) | 2.21 (2.02 to 2.39) |
| Pakistan | 423 (285 to 647) | 0.84 (0.57 to 1.29) | 1472 (1081 to 2066) | 1.48 (1.06 to 2.04) | 1.62 (1.34 to 1.9) |
| Southeast Asia, East Asia, and Oceania | |  |  |  |  |
| East Asia |  |  |  |  |  |
| China | 2648 (2088 to 3416) | 0.33 (0.27 to 0.42) | 9033 (7053 to 11644) | 0.46 (0.36 to 0.59) | 1.22 (1.08 to 1.36) |
| Democratic People's Republic of Korea | 87 (63 to 118) | 0.55 (0.41 to 0.73) | 175 (131 to 232) | 0.55 (0.41 to 0.72) | 0.13 (0.04 to 0.22) |
| Taiwan (Province of China) | 201 (190 to 212) | 1.35 (1.26 to 1.43) | 991 (888 to 1071) | 2.37 (2.14 to 2.56) | 0.79 (0.08 to 1.51) |
| Oceania |  |  |  |  |  |
| American Samoa | 1 (1 to 1) | 3.87 (3.25 to 4.55) | 1 (1 to 1) | 2.72 (2.24 to 3.3) | -1.47 (-1.63 to -1.31) |
| Cook Islands | 0 (0 to 0) | 3.52 (2.88 to 4.3) | 1 (0 to 1) | 2.94 (1.7 to 4.97) | -0.69 (-0.75 to -0.63) |
| Fiji | 10 (8 to 12) | 3.49 (2.89 to 4.17) | 21 (16 to 27) | 3.65 (2.86 to 4.58) | -0.19 (-0.39 to 0) |
| Guam | 4 (3 to 5) | 6.96 (5.93 to 8.22) | 4 (3 to 4) | 1.78 (1.51 to 2.07) | -4.2 (-4.41 to -3.99) |
| Kiribati | 0 (0 to 0) | 0.41 (0.34 to 0.49) | 0 (0 to 0) | 0.45 (0.34 to 0.58) | 0.19 (0.1 to 0.27) |
| Marshall Islands | 0 (0 to 1) | 3.17 (2.18 to 4.32) | 1 (1 to 1) | 2.92 (2.03 to 3.99) | -0.46 (-0.5 to -0.41) |
| Micronesia (Federated States of) | 2 (1 to 2) | 4.17 (3.18 to 5.52) | 2 (1 to 3) | 3.51 (2.73 to 4.51) | -0.75 (-0.85 to -0.65) |
| Nauru | 0 (0 to 0) | 4.92 (3.95 to 6.24) | 0 (0 to 0) | 6.01 (3.5 to 9.06) | 0.59 (0.55 to 0.62) |
| Niue | 0 (0 to 0) | 3.72 (2.9 to 4.63) | 0 (0 to 0) | 3.27 (2.58 to 3.98) | -0.65 (-0.73 to -0.57) |
| Northern Mariana Islands | 1 (1 to 1) | 5.54 (4.5 to 6.87) | 1 (1 to 2) | 3.38 (2.76 to 4.11) | -2.63 (-2.94 to -2.32) |
| Palau | 0 (0 to 0) | 3.34 (2.56 to 4.28) | 0 (0 to 1) | 2.93 (2.24 to 3.79) | -0.48 (-0.5 to -0.45) |
| Papua New Guinea | 22 (14 to 34) | 1.51 (0.94 to 2.31) | 66 (44 to 96) | 1.58 (1.07 to 2.25) | 0.04 (-0.05 to 0.12) |
| Samoa | 2 (2 to 3) | 3.4 (2.43 to 4.43) | 4 (3 to 5) | 2.8 (2.12 to 3.57) | -0.75 (-0.85 to -0.65) |
| Solomon Islands | 2 (1 to 3) | 1.77 (1.19 to 2.66) | 5 (3 to 7) | 1.97 (1.35 to 2.84) | 0.22 (0.12 to 0.32) |
| Tokelau | 0 (0 to 0) | 3.42 (2.63 to 4.38) | 0 (0 to 0) | 3.41 (2.24 to 4.78) | -0.23 (-0.3 to -0.16) |
| Tonga | 1 (1 to 2) | 2.87 (2.25 to 3.66) | 2 (2 to 3) | 3.04 (2.18 to 3.99) | 0.06 (-0.11 to 0.23) |
| Tuvalu | 0 (0 to 0) | 3.17 (2.16 to 4.48) | 0 (0 to 0) | 3.02 (2.44 to 3.75) | -0.33 (-0.4 to -0.26) |
| Vanuatu | 1 (1 to 2) | 2.19 (1.5 to 3.19) | 3 (2 to 4) | 2.23 (1.65 to 2.95) | -0.1 (-0.19 to -0.01) |
| Southeast Asia |  |  |  |  |  |
| Cambodia | 20 (12 to 32) | 0.55 (0.32 to 0.87) | 74 (47 to 119) | 0.75 (0.47 to 1.21) | 1.03 (0.91 to 1.16) |
| Indonesia | 497 (328 to 674) | 0.63 (0.41 to 0.87) | 1820 (1246 to 2446) | 1.03 (0.71 to 1.36) | 1.45 (1.36 to 1.54) |
| Lao People's Democratic Republic | 13 (8 to 21) | 0.78 (0.51 to 1.22) | 34 (25 to 47) | 0.93 (0.68 to 1.28) | 0.54 (0.51 to 0.57) |
| Malaysia | 268 (217 to 323) | 3.3 (2.66 to 4) | 971 (812 to 1159) | 4.04 (3.38 to 4.88) | 0.34 (0.03 to 0.65) |
| Maldives | 0 (0 to 1) | 0.66 (0.47 to 0.91) | 2 (1 to 3) | 0.6 (0.31 to 0.97) | -0.79 (-0.96 to -0.62) |
| Mauritius | 9 (9 to 10) | 1.5 (1.4 to 1.6) | 15 (14 to 16) | 0.88 (0.81 to 0.94) | -1.43 (-1.86 to -1) |
| Myanmar | 150 (88 to 225) | 0.8 (0.47 to 1.16) | 392 (296 to 514) | 0.96 (0.73 to 1.26) | 0.55 (0.51 to 0.59) |
| Philippines | 292 (251 to 337) | 1.22 (1.07 to 1.39) | 950 (779 to 1119) | 1.35 (1.12 to 1.58) | 0.4 (0.34 to 0.46) |
| Seychelles | 1 (1 to 1) | 1.08 (0.88 to 1.29) | 1 (1 to 2) | 1.07 (0.74 to 1.51) | 0.08 (-0.1 to 0.25) |
| Sri Lanka | 30 (24 to 36) | 0.35 (0.29 to 0.42) | 88 (59 to 122) | 0.37 (0.25 to 0.5) | 0.65 (0.43 to 0.87) |
| Thailand | 546 (418 to 723) | 2.02 (1.53 to 2.71) | 2134 (1640 to 2755) | 2.01 (1.55 to 2.58) | -0.46 (-0.61 to -0.31) |
| Timor-Leste | 1 (1 to 2) | 0.54 (0.38 to 0.82) | 5 (4 to 8) | 0.74 (0.49 to 1.15) | 1.12 (1 to 1.25) |
| Viet Nam | 238 (172 to 324) | 0.67 (0.48 to 0.9) | 895 (656 to 1193) | 1.07 (0.78 to 1.42) | 1.64 (1.56 to 1.71) |
| Sub-Saharan Africa |  |  |  |  |  |
| Central Sub-Saharan Africa |  |  |  |  |  |
| Angola | 82 (46 to 128) | 2.62 (1.48 to 4.12) | 273 (162 to 409) | 2.89 (1.72 to 4.26) | 0.18 (0.09 to 0.27) |
| Central African Republic | 24 (14 to 41) | 2.65 (1.51 to 4.65) | 37 (19 to 68) | 2.14 (1.08 to 3.73) | -0.89 (-0.98 to -0.8) |
| Congo | 35 (22 to 52) | 3.94 (2.46 to 5.74) | 74 (44 to 111) | 3.38 (2.01 to 5.05) | -0.79 (-0.99 to -0.59) |
| Democratic Republic of the Congo | 309 (157 to 552) | 2.53 (1.27 to 4.45) | 626 (309 to 1115) | 2.14 (1.08 to 3.82) | -0.71 (-1.05 to -0.37) |
| Equatorial Guinea | 4 (2 to 7) | 2.66 (1.55 to 4.37) | 15 (7 to 26) | 3.39 (1.69 to 5.75) | 0.73 (0.64 to 0.82) |
| Gabon | 22 (14 to 32) | 4.34 (2.63 to 6.17) | 33 (20 to 48) | 3.79 (2.35 to 5.45) | -0.72 (-0.85 to -0.59) |
| Eastern Sub-Saharan Africa |  |  |  |  |  |
| Burundi | 51 (29 to 83) | 2.41 (1.4 to 3.92) | 57 (25 to 106) | 1.44 (0.62 to 2.67) | -2.19 (-2.55 to -1.83) |
| Comoros | 4 (2 to 7) | 2.36 (0.95 to 4.32) | 8 (3 to 15) | 1.97 (0.74 to 3.6) | -0.96 (-1.17 to -0.75) |
| Djibouti | 2 (1 to 4) | 2.24 (1.15 to 3.43) | 10 (4 to 16) | 1.94 (0.86 to 3.09) | -0.76 (-0.89 to -0.62) |
| Eritrea | 17 (9 to 33) | 1.8 (0.96 to 3.24) | 42 (19 to 82) | 1.9 (0.84 to 3.58) | -0.19 (-0.34 to -0.03) |
| Ethiopia | 196 (118 to 357) | 1.2 (0.71 to 2.23) | 424 (219 to 720) | 1.1 (0.57 to 1.87) | -0.57 (-0.81 to -0.34) |
| Kenya | 103 (67 to 149) | 1.45 (0.92 to 2.11) | 337 (198 to 468) | 1.78 (1.05 to 2.49) | 0.54 (0.46 to 0.61) |
| Madagascar | 141 (74 to 243) | 3.07 (1.57 to 5.3) | 244 (119 to 403) | 2.53 (1.19 to 4.29) | -0.8 (-0.99 to -0.6) |
| Malawi | 48 (24 to 91) | 1.49 (0.71 to 2.77) | 122 (59 to 209) | 1.85 (0.93 to 3.18) | 0.47 (0.33 to 0.61) |
| Mozambique | 91 (39 to 179) | 1.86 (0.82 to 3.57) | 233 (94 to 454) | 2.4 (1.03 to 4.55) | 0.99 (0.92 to 1.07) |
| Rwanda | 67 (40 to 106) | 2.76 (1.67 to 4.31) | 96 (56 to 157) | 1.84 (1.12 to 3.04) | -2.01 (-2.38 to -1.63) |
| Somalia | 24 (13 to 53) | 1.27 (0.66 to 2.6) | 44 (20 to 106) | 0.86 (0.41 to 2.05) | -1.38 (-1.51 to -1.24) |
| South Sudan | 45 (18 to 83) | 1.93 (0.82 to 3.55) | 45 (20 to 89) | 1.39 (0.62 to 2.68) | -1.38 (-1.59 to -1.17) |
| Uganda | 86 (40 to 172) | 1.55 (0.75 to 3.04) | 205 (99 to 338) | 1.6 (0.8 to 2.64) | -0.22 (-0.37 to -0.08) |
| United Republic of Tanzania | 205 (123 to 343) | 2.23 (1.32 to 3.73) | 562 (271 to 957) | 2.5 (1.21 to 4.29) | -0.04 (-0.2 to 0.13) |
| Zambia | 53 (34 to 81) | 2.23 (1.44 to 3.35) | 205 (87 to 372) | 3.36 (1.53 to 5.94) | 1.46 (1.04 to 1.89) |
| Southern Sub-Saharan Africa |  |  |  |  |  |
| Botswana | 11 (7 to 17) | 2.57 (1.68 to 3.86) | 25 (16 to 35) | 2.22 (1.36 to 3.04) | -0.69 (-0.87 to -0.51) |
| Eswatini | 6 (4 to 8) | 2.43 (1.64 to 3.48) | 10 (7 to 15) | 2.21 (1.47 to 3.03) | -0.19 (-0.44 to 0.06) |
| Lesotho | 12 (7 to 18) | 1.63 (0.98 to 2.51) | 17 (9 to 27) | 1.96 (1.14 to 2.97) | 0.87 (0.69 to 1.06) |
| Namibia | 13 (8 to 19) | 2.55 (1.65 to 3.81) | 27 (20 to 38) | 2.48 (1.85 to 3.39) | -0.33 (-0.5 to -0.16) |
| South Africa | 591 (472 to 710) | 3.13 (2.45 to 3.82) | 935 (820 to 1052) | 2.29 (2.02 to 2.54) | -1.76 (-2.13 to -1.38) |
| Zimbabwe | 105 (85 to 127) | 3.35 (2.73 to 4.06) | 221 (164 to 297) | 3.96 (3.06 to 5.2) | 0.5 (0.34 to 0.67) |
| Western Sub-Saharan Africa |  |  |  |  |  |
| Benin | 29 (13 to 51) | 1.58 (0.75 to 2.79) | 70 (28 to 126) | 1.6 (0.65 to 2.86) | -0.12 (-0.23 to -0.01) |
| Burkina Faso | 60 (33 to 126) | 1.69 (0.91 to 3.53) | 155 (75 to 299) | 2.02 (0.98 to 3.85) | 0.75 (0.69 to 0.82) |
| Cabo Verde | 5 (2 to 9) | 1.88 (0.76 to 3.87) | 10 (5 to 18) | 2.46 (1.19 to 4.3) | 0.43 (0.16 to 0.71) |
| Cameroon | 88 (57 to 153) | 2.41 (1.54 to 4.29) | 247 (146 to 430) | 2.33 (1.42 to 3.97) | -0.44 (-0.58 to -0.3) |
| Chad | 35 (17 to 72) | 1.38 (0.66 to 2.83) | 73 (33 to 143) | 1.5 (0.66 to 2.99) | 0.06 (-0.05 to 0.17) |
| Cote d'Ivoire | 78 (37 to 140) | 2.54 (1.23 to 4.66) | 226 (100 to 393) | 2.44 (1.1 to 4.31) | -0.56 (-0.73 to -0.4) |
| Gambia | 7 (3 to 13) | 2.21 (0.95 to 4.24) | 21 (10 to 37) | 2.44 (1.12 to 4.26) | 0.02 (-0.13 to 0.18) |
| Ghana | 160 (80 to 267) | 3.1 (1.51 to 5.15) | 404 (184 to 683) | 2.87 (1.33 to 4.74) | -0.59 (-0.77 to -0.41) |
| Guinea | 50 (24 to 94) | 1.69 (0.8 to 3.2) | 93 (34 to 181) | 1.89 (0.7 to 3.66) | 0.17 (0.06 to 0.27) |
| Guinea-Bissau | 8 (5 to 15) | 2.45 (1.36 to 4.41) | 13 (7 to 24) | 2.24 (1.18 to 3.99) | -0.52 (-0.63 to -0.42) |
| Liberia | 22 (11 to 41) | 2.19 (1.15 to 4.02) | 34 (15 to 66) | 1.95 (0.86 to 3.7) | -0.44 (-0.6 to -0.28) |
| Mali | 40 (20 to 78) | 1.24 (0.63 to 2.52) | 88 (38 to 169) | 1.18 (0.51 to 2.27) | -0.19 (-0.41 to 0.04) |
| Mauritania | 24 (11 to 38) | 2.7 (1.18 to 4.35) | 44 (16 to 78) | 2.37 (0.85 to 4.15) | -0.84 (-1.03 to -0.64) |
| Niger | 28 (12 to 59) | 1.35 (0.58 to 2.85) | 76 (29 to 175) | 1.17 (0.45 to 2.69) | -0.61 (-0.74 to -0.49) |
| Nigeria | 956 (478 to 1774) | 2.61 (1.32 to 4.82) | 1691 (769 to 3088) | 2.3 (1.09 to 4.12) | -0.77 (-0.96 to -0.59) |
| Sao Tome and Principe | 1 (0 to 2) | 1.82 (0.85 to 3.38) | 2 (1 to 4) | 2.29 (1.14 to 4) | 0.8 (0.68 to 0.92) |
| Senegal | 60 (29 to 112) | 2.13 (1.02 to 3.99) | 143 (60 to 263) | 2.12 (0.9 to 3.88) | -0.32 (-0.44 to -0.19) |
| Sierra Leone | 40 (19 to 76) | 2.14 (1.04 to 4.04) | 63 (28 to 121) | 1.89 (0.82 to 3.62) | -0.58 (-0.68 to -0.49) |
| Togo | 24 (12 to 44) | 2.35 (1.13 to 4.29) | 71 (30 to 126) | 2.29 (0.96 to 4.18) | -0.42 (-0.55 to -0.29) |

UI, uncertainty intervals; EAPC, estimated annual percentage change; CI, confidence interval

**Table S4** The sex (male to female) ratio of age-standardized death rate for aortic aneurysm across 204 countries and territories in 2021

| **Location** | **Abbreviation of location** | **Sex ratio of age-standardized death rate (per 100,000) (95% UI)** |
| --- | --- | --- |
| Central Europe, Eastern Europe, and Central Asia |  | 2.88 (2.51 to 3.3) |
| Central Asia |  | 2.52 (2.13 to 2.99) |
| Armenia | ARM | 2.36 (1.8 to 3.11) |
| Azerbaijan | AZE | 2.52 (1.3 to 4.9) |
| Georgia | GEO | 5.02 (3.69 to 6.83) |
| Kazakhstan | KAZ | 2.87 (2.1 to 3.91) |
| Kyrgyzstan | KGZ | 2.7 (2.01 to 3.61) |
| Mongolia | MNG | 1.51 (0.9 to 2.53) |
| Tajikistan | TJK | 5.23 (3.17 to 8.61) |
| Turkmenistan | TKM | 2.53 (1.58 to 4.04) |
| Uzbekistan | UZB | 1.77 (1.3 to 2.42) |
| Central Europe |  | 2.9 (2.43 to 3.46) |
| Albania | ALB | 2.34 (1.31 to 4.16) |
| Bosnia and Herzegovina | BIH | 3.2 (1.89 to 5.41) |
| Bulgaria | BGR | 3.65 (2.6 to 5.12) |
| Croatia | HRV | 2.97 (2.27 to 3.89) |
| Czechia | CZE | 2.27 (1.79 to 2.88) |
| Hungary | HUN | 2.1 (1.72 to 2.55) |
| Montenegro | MNE | 3.44 (2.25 to 5.27) |
| North Macedonia | MKD | 2.8 (1.43 to 5.49) |
| Poland | POL | 3.34 (2.69 to 4.14) |
| Romania | ROU | 2.28 (1.75 to 2.98) |
| Serbia | SRB | 3.33 (2.27 to 4.88) |
| Slovakia | SVK | 2.9 (1.94 to 4.36) |
| Slovenia | SVN | 2.92 (2.03 to 4.2) |
| Eastern Europe |  | 3 (2.56 to 3.52) |
| Belarus | BLR | 4.15 (3.1 to 5.54) |
| Estonia | EST | 3.65 (2.9 to 4.6) |
| Latvia | LVA | 3.98 (3.03 to 5.24) |
| Lithuania | LTU | 3.51 (2.72 to 4.52) |
| Republic of Moldova | MDA | 2.91 (2.35 to 3.59) |
| Russian Federation | RUS | 2.82 (2.39 to 3.32) |
| Ukraine | UKR | 3.89 (2.3 to 6.58) |
| High-income |  | 2.08 (1.76 to 2.47) |
| Australasia |  | 1.94 (1.62 to 2.31) |
| Australia | AUS | 2.02 (1.68 to 2.43) |
| New Zealand | NZL | 1.69 (1.42 to 2.02) |
| High-income Asia Pacific |  | 1.72 (1.4 to 2.13) |
| Brunei Darussalam | BRN | 1.92 (1.33 to 2.78) |
| Japan | JPN | 1.78 (1.45 to 2.18) |
| Republic of Korea | KOR | 1.66 (1.14 to 2.41) |
| Singapore | SGP | 1.85 (1.54 to 2.21) |
| High-income North America |  | 1.92 (1.68 to 2.19) |
| Canada | CAN | 2.03 (1.71 to 2.41) |
| Greenland | GRL | 1.7 (1.13 to 2.57) |
| United States of America | USA | 1.9 (1.67 to 2.17) |
| Southern Latin America |  | 2.99 (2.64 to 3.4) |
| Argentina | ARG | 3.51 (3.05 to 4.03) |
| Chile | CHL | 2.1 (1.81 to 2.43) |
| Uruguay | URY | 2.79 (2.41 to 3.24) |
| Western Europe |  | 2.63 (2.26 to 3.06) |
| Andorra | AND | 2.59 (1.35 to 4.97) |
| Austria | AUT | 2.03 (1.71 to 2.42) |
| Belgium | BEL | 3.19 (2.63 to 3.87) |
| Cyprus | CYP | 3.5 (2.42 to 5.06) |
| Denmark | DNK | 2.29 (1.93 to 2.72) |
| Finland | FIN | 2.65 (2.2 to 3.2) |
| France | FRA | 3.11 (2.57 to 3.77) |
| Germany | DEU | 2.37 (2 to 2.8) |
| Greece | GRC | 3.91 (3.36 to 4.56) |
| Iceland | ISL | 1.63 (1.32 to 2) |
| Ireland | IRL | 1.67 (1.32 to 2.1) |
| Israel | ISR | 2.39 (1.97 to 2.91) |
| Italy | ITA | 3.21 (2.59 to 3.99) |
| Luxembourg | LUX | 3.34 (2.78 to 4.02) |
| Malta | MLT | 4.46 (3.66 to 5.43) |
| Monaco | MCO | 1.55 (0.95 to 2.51) |
| Netherlands | NLD | 2.37 (1.96 to 2.88) |
| Norway | NOR | 1.83 (1.56 to 2.14) |
| Portugal | PRT | 3.9 (3.27 to 4.65) |
| San Marino | SMR | 8.47 (4.36 to 16.46) |
| Spain | ESP | 5.02 (4.1 to 6.14) |
| Sweden | SWE | 1.56 (1.25 to 1.95) |
| Switzerland | CHE | 2.39 (1.89 to 3.01) |
| United Kingdom | GBR | 1.94 (1.67 to 2.24) |
| Latin America and Caribbean |  | 1.93 (1.7 to 2.18) |
| Andean Latin America |  | 1.98 (1.47 to 2.68) |
| Bolivia (Plurinational State of) | BOL | 1.85 (1.13 to 3.03) |
| Ecuador | ECU | 1.94 (1.3 to 2.89) |
| Peru | PER | 2.14 (1.35 to 3.37) |
| Caribbean |  | 2.36 (1.95 to 2.87) |
| Antigua and Barbuda | ATG | 2.62 (2.23 to 3.08) |
| Bahamas | BHS | 1.52 (1.17 to 1.99) |
| Barbados | BRB | 2.56 (1.88 to 3.48) |
| Belize | BLZ | 1.03 (0.84 to 1.28) |
| Bermuda | BMU | 3.83 (2.9 to 5.06) |
| Cuba | CUB | 2.59 (2.09 to 3.21) |
| Dominica | DMA | 1.46 (1.01 to 2.11) |
| Dominican Republic | DOM | 2.61 (1.6 to 4.25) |
| Grenada | GRD | 2.6 (2.19 to 3.08) |
| Guyana | GUY | 1.62 (1.13 to 2.33) |
| Haiti | HTI | 1.57 (0.63 to 3.93) |
| Jamaica | JAM | 1.72 (1.2 to 2.47) |
| Puerto Rico | PRI | 2.54 (1.95 to 3.3) |
| Saint Kitts and Nevis | KNA | 2.56 (1.98 to 3.31) |
| Saint Lucia | LCA | 1.34 (1.05 to 1.71) |
| Saint Vincent and the Grenadines | VCT | 2.56 (2.11 to 3.1) |
| Suriname | SUR | 3.89 (2.35 to 6.43) |
| Trinidad and Tobago | TTO | 2.24 (1.58 to 3.18) |
| United States Virgin Islands | VIR | 2.62 (1.49 to 4.63) |
| Central Latin America |  | 2.4 (1.96 to 2.94) |
| Colombia | COL | 2.52 (1.92 to 3.32) |
| Costa Rica | CRI | 3.2 (2.63 to 3.89) |
| El Salvador | SLV | 3.24 (2.16 to 4.85) |
| Guatemala | GTM | 2.02 (1.62 to 2.53) |
| Honduras | HND | 1.95 (1.12 to 3.37) |
| Mexico | MEX | 2.41 (1.9 to 3.07) |
| Nicaragua | NIC | 2.75 (1.95 to 3.88) |
| Panama | PAN | 2.52 (1.83 to 3.49) |
| Venezuela (Bolivarian Republic of) | VEN | 2.29 (1.57 to 3.33) |
| Tropical Latin America |  | 1.8 (1.59 to 2.04) |
| Brazil | BRA | 1.79 (1.58 to 2.03) |
| Paraguay | PRY | 2.83 (1.84 to 4.36) |
| North Africa and Middle East |  | 2.62 (2.05 to 3.35) |
| Afghanistan | AFG | 2.51 (0.94 to 6.67) |
| Algeria | DZA | 1.94 (1.21 to 3.1) |
| Bahrain | BHR | 2.09 (1.43 to 3.06) |
| Egypt | EGY | 2.97 (2.1 to 4.19) |
| Iran (Islamic Republic of) | IRN | 2.69 (2.16 to 3.36) |
| Iraq | IRQ | 2.66 (1.7 to 4.16) |
| Jordan | JOR | 2.91 (1.92 to 4.43) |
| Kuwait | KWT | 4.38 (3.32 to 5.8) |
| Lebanon | LBN | 3.54 (2.55 to 4.9) |
| Libya | LBY | 2.71 (1.33 to 5.52) |
| Morocco | MAR | 2.61 (1.51 to 4.53) |
| Oman | OMN | 4.32 (2.38 to 7.84) |
| Palestine | PSE | 3.88 (2.52 to 5.97) |
| Qatar | QAT | 2.27 (1.2 to 4.27) |
| Saudi Arabia | SAU | 0.83 (0.46 to 1.5) |
| Sudan | SDN | 2.52 (1.27 to 5.03) |
| Syrian Arab Republic | SYR | 2.75 (1.8 to 4.19) |
| Tunisia | TUN | 3.45 (1.72 to 6.91) |
| Turkiye | TUR | 3.2 (2.17 to 4.72) |
| United Arab Emirates | ARE | 0.32 (0.21 to 0.48) |
| Yemen | YEM | 2.36 (1.07 to 5.23) |
| South Asia |  | 1.74 (0.94 to 3.2) |
| Bangladesh | BGD | 1.6 (0.71 to 3.62) |
| Bhutan | BTN | 1.5 (0.69 to 3.25) |
| India | IND | 1.79 (0.94 to 3.42) |
| Nepal | NPL | 1.3 (0.59 to 2.87) |
| Pakistan | PAK | 1.37 (0.72 to 2.59) |
| Southeast Asia, East Asia, and Oceania |  | 2.52 (1.96 to 3.25) |
| East Asia |  | 2.95 (2.02 to 4.33) |
| China | CHN | 2.96 (1.92 to 4.57) |
| Democratic People's Republic of Korea | PRK | 1.89 (1.18 to 3.01) |
| Taiwan (Province of China) | TWN | 3.12 (2.64 to 3.7) |
| Oceania |  | 1.06 (0.68 to 1.66) |
| American Samoa | ASM | 1.07 (0.76 to 1.51) |
| Cook Islands | COK | 1.57 (0.61 to 4.08) |
| Fiji | FJI | 1.61 (1.1 to 2.35) |
| Guam | GUM | 2.79 (2.09 to 3.74) |
| Kiribati | KIR | 2.29 (1.41 to 3.72) |
| Marshall Islands | MHL | 1.07 (0.58 to 2.01) |
| Micronesia (Federated States of) | FSM | 1.03 (0.67 to 1.59) |
| Nauru | NRU | 0.97 (0.47 to 2.02) |
| Niue | GUM | 1.44 (0.94 to 2.21) |
| Northern Mariana Islands | MNP | 0.9 (0.65 to 1.26) |
| Palau | PLW | 0.97 (0.6 to 1.57) |
| Papua New Guinea | PNG | 0.91 (0.42 to 1.96) |
| Samoa | WSM | 1.16 (0.71 to 1.89) |
| Solomon Islands | SLB | 0.94 (0.45 to 2) |
| Tokelau | TKL | 0.96 (0.51 to 1.8) |
| Tonga | TON | 2.33 (1.4 to 3.87) |
| Tuvalu | TUV | 1.05 (0.7 to 1.6) |
| Vanuatu | VUT | 1.15 (0.64 to 2.05) |
| Southeast Asia |  | 2.17 (1.68 to 2.79) |
| Cambodia | KHM | 2.27 (1.09 to 4.75) |
| Indonesia | IDN | 1.7 (0.89 to 3.26) |
| Lao People's Democratic Republic | LAO | 1.88 (1.11 to 3.21) |
| Malaysia | MYS | 2.68 (1.93 to 3.72) |
| Maldives | MDV | 2.16 (0.92 to 5.08) |
| Mauritius | MUS | 3.36 (2.91 to 3.87) |
| Myanmar | MMR | 2.44 (1.5 to 3.96) |
| Philippines | PHL | 1.93 (1.38 to 2.71) |
| Seychelles | SYC | 2.61 (1.39 to 4.89) |
| Sri Lanka | LKA | 3.38 (1.96 to 5.83) |
| Thailand | THA | 2.07 (1.37 to 3.13) |
| Timor-Leste | TLS | 1.58 (0.79 to 3.18) |
| Viet Nam | VNM | 2.88 (1.73 to 4.77) |
| Sub-Saharan Africa |  | 1.89 (0.93 to 3.85) |
| Central Sub-Saharan Africa |  | 1.62 (0.68 to 3.86) |
| Angola | AGO | 1.78 (0.87 to 3.65) |
| Central African Republic | CAF | 1.55 (0.52 to 4.65) |
| Congo | COG | 1.8 (0.89 to 3.65) |
| Democratic Republic of the Congo | COD | 1.48 (0.52 to 4.25) |
| Equatorial Guinea | GNQ | 2.43 (0.97 to 6.08) |
| Gabon | GAB | 2.12 (1.09 to 4.11) |
| Eastern Sub-Saharan Africa |  | 1.78 (0.77 to 4.11) |
| Burundi | BDI | 1.54 (0.46 to 5.09) |
| Comoros | COM | 1.83 (0.53 to 6.36) |
| Djibouti | DJI | 1.77 (0.63 to 4.96) |
| Eritrea | ERI | 1.54 (0.5 to 4.77) |
| Ethiopia | ETH | 1.43 (0.54 to 3.75) |
| Kenya | KEN | 1.7 (0.85 to 3.4) |
| Madagascar | MDG | 1.66 (0.63 to 4.37) |
| Malawi | MWI | 2.59 (0.97 to 6.93) |
| Mozambique | MOZ | 2.19 (0.69 to 7) |
| Rwanda | RWA | 1.73 (0.7 to 4.23) |
| Somalia | SOM | 0.99 (0.15 to 6.44) |
| South Sudan | SSD | 1.63 (0.44 to 6.08) |
| Uganda | UGA | 2.1 (0.8 to 5.51) |
| United Republic of Tanzania | TZA | 2.15 (0.86 to 5.37) |
| Zambia | ZMB | 1.92 (0.71 to 5.17) |
| Southern Sub-Saharan Africa |  | 2.49 (2.1 to 2.94) |
| Botswana | BWA | 2.39 (1.22 to 4.69) |
| Eswatini | SWZ | 2.57 (1.39 to 4.76) |
| Lesotho | LSO | 1.77 (0.71 to 4.38) |
| Namibia | NAM | 2.51 (1.42 to 4.42) |
| South Africa | ZAF | 2.65 (2.12 to 3.32) |
| Zimbabwe | ZWE | 1.79 (1.11 to 2.88) |
| Western Sub-Saharan Africa |  | 1.89 (0.74 to 4.83) |
| Benin | BEN | 2.16 (0.66 to 7.03) |
| Burkina Faso | BFA | 1.7 (0.5 to 5.83) |
| Cabo Verde | CPV | 2.86 (1.08 to 7.6) |
| Cameroon | CMR | 1.83 (0.83 to 4.05) |
| Chad | TCD | 1.27 (0.33 to 4.92) |
| Cote d'Ivoire | CIV | 2.14 (0.78 to 5.86) |
| Gambia | GMB | 2.05 (0.74 to 5.73) |
| Ghana | GHA | 2.21 (0.87 to 5.62) |
| Guinea | GIN | 1.57 (0.44 to 5.56) |
| Guinea-Bissau | GNB | 1.79 (0.64 to 4.99) |
| Liberia | LBR | 1.57 (0.44 to 5.64) |
| Mali | MLI | 1 (0.24 to 4.09) |
| Mauritania | MRT | 1.53 (0.44 to 5.24) |
| Niger | NER | 0.88 (0.15 to 5.06) |
| Nigeria | NGA | 2.04 (0.74 to 5.63) |
| Sao Tome and Principe | STP | 1.67 (0.65 to 4.3) |
| Senegal | SEN | 1.79 (0.55 to 5.82) |
| Sierra Leone | SLE | 1.77 (0.55 to 5.72) |
| Togo | TGO | 2.12 (0.7 to 6.4) |

UI, uncertainty intervals

**Table S5** The socio-demographic index across 204 countries and territories in 2021

| **Location** | **Abbreviation of location** | **Socio-demographic Index (SDI) in 1990** | **Socio-demographic Index (SDI) in 2021** |
| --- | --- | --- | --- |
| Central Europe, Eastern Europe, and Central Asia |  | 0.638 | 0.769 |
| Central Asia |  | 0.553 | 0.675 |
| Armenia | ARM | 0.544 | 0.702 |
| Azerbaijan | AZE | 0.596 | 0.695 |
| Georgia | GEO | 0.656 | 0.732 |
| Kazakhstan | KAZ | 0.589 | 0.725 |
| Kyrgyzstan | KGZ | 0.519 | 0.604 |
| Mongolia | MNG | 0.467 | 0.618 |
| Tajikistan | TJK | 0.466 | 0.542 |
| Turkmenistan | TKM | 0.563 | 0.682 |
| Uzbekistan | UZB | 0.500 | 0.663 |
| Central Europe |  | 0.637 | 0.796 |
| Albania | ALB | 0.558 | 0.707 |
| Bosnia and Herzegovina | BIH | 0.541 | 0.723 |
| Bulgaria | BGR | 0.633 | 0.768 |
| Croatia | HRV | 0.669 | 0.798 |
| Czechia | CZE | 0.682 | 0.828 |
| Hungary | HUN | 0.649 | 0.791 |
| Montenegro | MNE | 0.674 | 0.796 |
| North Macedonia | MKD | 0.609 | 0.751 |
| Poland | POL | 0.627 | 0.812 |
| Romania | ROU | 0.619 | 0.768 |
| Serbia | SRB | 0.631 | 0.792 |
| Slovakia | SVK | 0.654 | 0.811 |
| Slovenia | SVN | 0.727 | 0.842 |
| Eastern Europe |  | 0.664 | 0.803 |
| Belarus | BLR | 0.622 | 0.784 |
| Estonia | EST | 0.675 | 0.845 |
| Latvia | LVA | 0.680 | 0.831 |
| Lithuania | LTU | 0.669 | 0.856 |
| Republic of Moldova | MDA | 0.604 | 0.732 |
| Russian Federation | RUS | 0.672 | 0.809 |
| Ukraine | UKR | 0.647 | 0.761 |
| High-income |  | 0.752 | 0.852 |
| Australasia |  | 0.731 | 0.846 |
| Australia | AUS | 0.726 | 0.844 |
| New Zealand | NZL | 0.752 | 0.849 |
| High-income Asia Pacific |  | 0.768 | 0.877 |
| Brunei Darussalam | BRN | 0.666 | 0.810 |
| Japan | JPN | 0.790 | 0.871 |
| Republic of Korea | KOR | 0.692 | 0.887 |
| Singapore | SGP | 0.686 | 0.856 |
| High-income North America |  | 0.766 | 0.863 |
| Canada | CAN | 0.782 | 0.873 |
| Greenland | GRL | 0.732 | 0.826 |
| United States of America | USA | 0.764 | 0.862 |
| Southern Latin America |  | 0.587 | 0.736 |
| Argentina | ARG | 0.587 | 0.723 |
| Chile | CHL | 0.586 | 0.772 |
| Uruguay | URY | 0.582 | 0.719 |
| Western Europe |  | 0.746 | 0.849 |
| Andorra | AND | 0.761 | 0.869 |
| Austria | AUT | 0.750 | 0.854 |
| Belgium | BEL | 0.737 | 0.854 |
| Cyprus | CYP | 0.648 | 0.836 |
| Denmark | DNK | 0.801 | 0.896 |
| Finland | FIN | 0.756 | 0.860 |
| France | FRA | 0.731 | 0.838 |
| Germany | DEU | 0.817 | 0.903 |
| Greece | GRC | 0.674 | 0.792 |
| Iceland | ISL | 0.764 | 0.876 |
| Ireland | IRL | 0.720 | 0.874 |
| Israel | ISR | 0.709 | 0.809 |
| Italy | ITA | 0.706 | 0.806 |
| Luxembourg | LUX | 0.781 | 0.884 |
| Malta | MLT | 0.657 | 0.802 |
| Monaco | MCO | 0.845 | 0.908 |
| Netherlands | NLD | 0.795 | 0.888 |
| Norway | NOR | 0.796 | 0.916 |
| Portugal | PRT | 0.600 | 0.744 |
| San Marino | SMR | 0.813 | 0.888 |
| Spain | ESP | 0.637 | 0.769 |
| Sweden | SWE | 0.786 | 0.887 |
| Switzerland | CHE | 0.863 | 0.933 |
| United Kingdom | GBR | 0.744 | 0.859 |
| Latin America and Caribbean |  | 0.498 | 0.647 |
| Andean Latin America |  | 0.500 | 0.652 |
| Bolivia (Plurinational State of) | BOL | 0.424 | 0.599 |
| Ecuador | ECU | 0.518 | 0.661 |
| Peru | PER | 0.510 | 0.662 |
| Caribbean |  | 0.518 | 0.642 |
| Antigua and Barbuda | ATG | 0.612 | 0.750 |
| Bahamas | BHS | 0.694 | 0.805 |
| Barbados | BRB | 0.654 | 0.747 |
| Belize | BLZ | 0.424 | 0.610 |
| Bermuda | BMU | 0.696 | 0.821 |
| Cuba | CUB | 0.558 | 0.669 |
| Dominica | DMA | 0.564 | 0.747 |
| Dominican Republic | DOM | 0.443 | 0.619 |
| Grenada | GRD | 0.437 | 0.669 |
| Guyana | GUY | 0.460 | 0.651 |
| Haiti | HTI | 0.310 | 0.448 |
| Jamaica | JAM | 0.535 | 0.683 |
| Puerto Rico | PRI | 0.659 | 0.826 |
| Saint Kitts and Nevis | KNA | 0.581 | 0.755 |
| Saint Lucia | LCA | 0.496 | 0.673 |
| Saint Vincent and the Grenadines | VCT | 0.476 | 0.637 |
| Suriname | SUR | 0.502 | 0.634 |
| Trinidad and Tobago | TTO | 0.624 | 0.769 |
| United States Virgin Islands | VIR | 0.655 | 0.822 |
| Central Latin America |  | 0.486 | 0.641 |
| Colombia | COL | 0.481 | 0.655 |
| Costa Rica | CRI | 0.534 | 0.700 |
| El Salvador | SLV | 0.373 | 0.564 |
| Guatemala | GTM | 0.312 | 0.540 |
| Honduras | HND | 0.332 | 0.513 |
| Mexico | MEX | 0.505 | 0.665 |
| Nicaragua | NIC | 0.346 | 0.524 |
| Panama | PAN | 0.546 | 0.709 |
| Venezuela (Bolivarian Republic of) | VEN | 0.517 | 0.597 |
| Tropical Latin America |  | 0.500 | 0.652 |
| Brazil | BRA | 0.500 | 0.653 |
| Paraguay | PRY | 0.470 | 0.636 |
| North Africa and Middle East |  | 0.437 | 0.658 |
| Afghanistan | AFG | 0.174 | 0.337 |
| Algeria | DZA | 0.460 | 0.660 |
| Bahrain | BHR | 0.585 | 0.753 |
| Egypt | EGY | 0.417 | 0.607 |
| Iran (Islamic Republic of) | IRN | 0.454 | 0.697 |
| Iraq | IRQ | 0.412 | 0.663 |
| Jordan | JOR | 0.539 | 0.725 |
| Kuwait | KWT | 0.665 | 0.847 |
| Lebanon | LBN | 0.537 | 0.745 |
| Libya | LBY | 0.528 | 0.726 |
| Morocco | MAR | 0.358 | 0.563 |
| Oman | OMN | 0.429 | 0.773 |
| Palestine | PSE | 0.402 | 0.631 |
| Qatar | QAT | 0.651 | 0.847 |
| Saudi Arabia | SAU | 0.539 | 0.815 |
| Sudan | SDN | 0.292 | 0.542 |
| Syrian Arab Republic | SYR | 0.430 | 0.623 |
| Tunisia | TUN | 0.471 | 0.682 |
| Turkiye | TUR | 0.462 | 0.713 |
| United Arab Emirates | ARE | 0.644 | 0.849 |
| Yemen | YEM | 0.216 | 0.450 |
| South Asia |  | 0.320 | 0.558 |
| Bangladesh | BGD | 0.229 | 0.492 |
| Bhutan | BTN | 0.215 | 0.473 |
| India | IND | 0.333 | 0.575 |
| Nepal | NPL | 0.200 | 0.433 |
| Pakistan | PAK | 0.310 | 0.504 |
| Southeast Asia, East Asia, and Oceania |  | 0.471 | 0.699 |
| East Asia |  | 0.471 | 0.726 |
| China | CHN | 0.459 | 0.722 |
| Democratic People's Republic of Korea | PRK | 0.498 | 0.570 |
| Taiwan (Province of China) | TWN | 0.668 | 0.875 |
| Oceania |  | 0.391 | 0.467 |
| American Samoa | ASM | 0.614 | 0.724 |
| Cook Islands | COK | 0.565 | 0.779 |
| Fiji | FJI | 0.535 | 0.675 |
| Guam | GUM | 0.676 | 0.804 |
| Kiribati | KIR | 0.410 | 0.527 |
| Marshall Islands | MHL | 0.431 | 0.574 |
| Micronesia (Federated States of) | FSM | 0.463 | 0.588 |
| Nauru | NRU | 0.539 | 0.625 |
| Niue | GUM | 0.588 | 0.726 |
| Northern Mariana Islands | MNP | 0.709 | 0.772 |
| Palau | PLW | 0.663 | 0.754 |
| Papua New Guinea | PNG | 0.311 | 0.418 |
| Samoa | WSM | 0.487 | 0.593 |
| Solomon Islands | SLB | 0.301 | 0.429 |
| Tokelau | TKL | 0.522 | 0.686 |
| Tonga | TON | 0.492 | 0.626 |
| Tuvalu | TUV | 0.406 | 0.577 |
| Vanuatu | VUT | 0.353 | 0.473 |
| Southeast Asia |  | 0.464 | 0.650 |
| Cambodia | KHM | 0.289 | 0.474 |
| Indonesia | IDN | 0.457 | 0.657 |
| Lao People's Democratic Republic | LAO | 0.264 | 0.489 |
| Malaysia | MYS | 0.546 | 0.743 |
| Maldives | MDV | 0.332 | 0.651 |
| Mauritius | MUS | 0.545 | 0.718 |
| Myanmar | MMR | 0.319 | 0.534 |
| Philippines | PHL | 0.510 | 0.651 |
| Seychelles | SYC | 0.576 | 0.730 |
| Sri Lanka | LKA | 0.523 | 0.702 |
| Thailand | THA | 0.507 | 0.683 |
| Timor-Leste | TLS | 0.262 | 0.445 |
| Viet Nam | VNM | 0.408 | 0.628 |
| Sub-Saharan Africa |  | 0.297 | 0.459 |
| Central Sub-Saharan Africa |  | 0.302 | 0.472 |
| Angola | AGO | 0.271 | 0.454 |
| Central African Republic | CAF | 0.217 | 0.309 |
| Congo | COG | 0.421 | 0.583 |
| Democratic Republic of the Congo | COD | 0.290 | 0.383 |
| Equatorial Guinea | GNQ | 0.269 | 0.658 |
| Gabon | GAB | 0.455 | 0.635 |
| Eastern Sub-Saharan Africa |  | 0.234 | 0.410 |
| Burundi | BDI | 0.206 | 0.289 |
| Comoros | COM | 0.270 | 0.476 |
| Djibouti | DJI | 0.338 | 0.488 |
| Eritrea | ERI | 0.216 | 0.404 |
| Ethiopia | ETH | 0.148 | 0.359 |
| Kenya | KEN | 0.334 | 0.524 |
| Madagascar | MDG | 0.280 | 0.400 |
| Malawi | MWI | 0.204 | 0.385 |
| Mozambique | MOZ | 0.173 | 0.326 |
| Rwanda | RWA | 0.275 | 0.436 |
| Somalia | SOM | 0.049 | 0.078 |
| South Sudan | SSD | 0.207 | 0.278 |
| Uganda | UGA | 0.187 | 0.423 |
| United Republic of Tanzania | TZA | 0.259 | 0.447 |
| Zambia | ZMB | 0.304 | 0.506 |
| Southern Sub-Saharan Africa |  | 0.507 | 0.642 |
| Botswana | BWA | 0.418 | 0.643 |
| Eswatini | SWZ | 0.399 | 0.585 |
| Lesotho | LSO | 0.339 | 0.510 |
| Namibia | NAM | 0.450 | 0.618 |
| South Africa | ZAF | 0.542 | 0.680 |
| Zimbabwe | ZWE | 0.399 | 0.474 |
| Western Sub-Saharan Africa |  | 0.274 | 0.446 |
| Benin | BEN | 0.219 | 0.373 |
| Burkina Faso | BFA | 0.130 | 0.285 |
| Cabo Verde | CPV | 0.277 | 0.534 |
| Cameroon | CMR | 0.303 | 0.480 |
| Chad | TCD | 0.115 | 0.240 |
| Cote d'Ivoire | CIV | 0.279 | 0.426 |
| Gambia | GMB | 0.239 | 0.410 |
| Ghana | GHA | 0.373 | 0.565 |
| Guinea | GIN | 0.178 | 0.336 |
| Guinea-Bissau | GNB | 0.208 | 0.353 |
| Liberia | LBR | 0.235 | 0.352 |
| Mali | MLI | 0.127 | 0.269 |
| Mauritania | MRT | 0.336 | 0.499 |
| Niger | NER | 0.081 | 0.168 |
| Nigeria | NGA | 0.306 | 0.503 |
| Sao Tome and Principe | STP | 0.310 | 0.505 |
| Senegal | SEN | 0.238 | 0.408 |
| Sierra Leone | SLE | 0.212 | 0.359 |
| Togo | TGO | 0.270 | 0.409 |

**Table S6** Global number of population and EAPC in number of populations by age from 1990 to 2021

| **Age group** | **1990** | **2021** | **1999-2021** |
| --- | --- | --- | --- |
|  | **Number (×100,000) (95% UI)** | **Number (×100,000) (95% UI)** | **EAPC  (95% CI)** |
| All ages | 53336.23 (52310.36 to 54446.5) | 78913.53 (76667.34 to 81312.25) | 1.28 (1.27 to 1.29) |
| 15-19 years | 5194.23 (5088.63 to 5307.25) | 6239.8 (6046.17 to 6442.52) | 0.63 (0.49 to 0.77) |
| 20-24 years | 4920.88 (4815.25 to 5034.81) | 5971.58 (5787.49 to 6169.01) | 0.82 (0.69 to 0.96) |
| 25-29 years | 4426.2 (4336.2 to 4524.4) | 5883.43 (5704.87 to 6074.22) | 1 (0.9 to 1.1) |
| 30-34 years | 3854.22 (3778.87 to 3935.51) | 6044.8 (5861.95 to 6236.04) | 1.29 (1.18 to 1.4) |
| 35-39 years | 3522.44 (3449.23 to 3603.67) | 5608.66 (5442.52 to 5782.98) | 1.41 (1.28 to 1.54) |
| 40-44 years | 2864.82 (2808.6 to 2926.86) | 5002.51 (4859.28 to 5154.32) | 1.78 (1.62 to 1.94) |
| 45-49 years | 2321.95 (2277.25 to 2370.41) | 4735.05 (4602.13 to 4879.2) | 2.42 (2.26 to 2.57) |
| 50-54 years | 2125.71 (2085.65 to 2169.72) | 4449.23 (4323.01 to 4587.14) | 2.71 (2.59 to 2.84) |
| 55-59 years | 1852 (1816.3 to 1891.73) | 3957.28 (3847.65 to 4076.69) | 2.65 (2.47 to 2.82) |
| 60-64 years | 1606.09 (1576.24 to 1638.59) | 3200.48 (3115.24 to 3293.88) | 2.47 (2.26 to 2.68) |
| 65-69 years | 1236.09 (1213.83 to 1260.45) | 2758.42 (2681.17 to 2842.18) | 2.4 (2.21 to 2.6) |
| 70-74 years | 846.61 (831.01 to 863.21) | 2058.39 (2004.07 to 2119.72) | 2.49 (2.36 to 2.62) |
| 75-79 years | 615.56 (605.48 to 626.31) | 1318.84 (1285.19 to 1356.13) | 2.8 (2.68 to 2.92) |
| 80-84 years | 353.76 (348.1 to 359.6) | 875.83 (854.82 to 899.49) | 3.2 (3.05 to 3.34) |
| 85-89 years | 151.11 (148.84 to 153.5) | 457.22 (447.09 to 468.53) | 3.68 (3.53 to 3.84) |
| 90-94 years | 42.85 (42.19 to 43.55) | 178.89 (175.19 to 182.89) | 4.53 (4.38 to 4.67) |
| 95+ years | 10.18 (9.99 to 10.38) | 54.5 (53.37 to 55.7) | 5.66 (5.54 to 5.77) |

UI, uncertainty intervals; EAPC, estimated annual percentage change; CI, confidence interval

**Table S7** The population attributable fraction of risk factors for major vascular disease from 1990 to 2021

| **Risk factor** | **Population attributable fraction (PAF) (95% UI)** | | | |
| --- | --- | --- | --- | --- |
|  | **1990** | **2000** | **2010** | **2021** |
| Ischaemic heart disease |  |  |  |  |
| Smoking | 19.2 (16.6 to 21.8) | 17.9 (15.5 to 20.3) | 16.7 (14.3 to 19.0) | 15.0 (12.7 to 17.3) |
| High systolic blood pressure | 53.1 (43.6 to 61.3) | 52.9 (43.3 to 61.1) | 52.5 (43.0 to 60.9) | 52.2 (43.0 to 60.6) |
| High body-mass index | 9.11 (3.50 to 14.8) | 9.94 (3.81 to 16.1) | 10.2 (3.89 to 16.3) | 11.0 (4.20 to 17.5) |
| Diet high in sodium | 6.79 (1.38 to 16.4) | 6.81 (1.30 to 16.5) | 7.22 (1.33 to 17.3) | 7.33 (1.29 to 17.5) |
| Ischaemic stroke |  |  |  |  |
| Smoking | 10.9 (9.06 to 13.1) | 10.4 (8.72 to 12.3) | 10.2 (8.42 to 12.0) | 9.54 (7.76 to 11.6) |
| High systolic blood pressure | 58.9 (45.0 to 70.0) | 58.9 (44.8 to 70.4) | 58.8 (44.4 to 70.1) | 58.2 (44.0 to 69.3) |
| High body-mass index | 3.80 (0.547 to 7.46) | 4.20 (0.611 to 8.25) | 4.29 (0.628 to 8.40) | 4.80 (0.703 to 9.32) |
| Diet high in sodium | 8.90 (2.11 to 20.2) | 8.86 (1.97 to 20.0) | 9.07 (1.97 to 20.4) | 9.20 (1.91 to 20.9) |
| Lower extremity peripheral arterial disease |  |  |  |  |
| Smoking | 28.3 (22.0 to 34.6) | 26.7 (20.7 to 32.8) | 22.7 (17.4 to 28.2) | 20.0 (15.1 to 25.1) |
| High systolic blood pressure | 14.3 (2.97 to 25.4) | 14.1 (2.92 to 25.1) | 13.5 (2.79 to 24.1) | 13.1 (2.70 to 23.6) |
| High body-mass index | 14.5 (3.73 to 37.2) | 16.8 (4.45 to 41.3) | 18.0 (4.99 to 42.4) | 19.6 (5.61 to 44.2) |
| Diet high in sodium | 0.464 (0.0249 to 1.50) | 0.469 (0.0230 to 1.56) | 0.516 (0.0231 to 1.74) | 0.557 (0.0276 to 1.84) |

UI, uncertainty intervals
